# Comparison of size distribution and (Pro249-Ser258) epitope exposure in in vitro and in vivo derived Tau fibrils

**DOI:** 10.1186/s12860-020-00320-y

**Published:** 2020-11-12

**Authors:** André Marreiro, Kristof Van Kolen, Cristiano Sousa, Liesbet Temmerman, Bruno Vasconcelos, Rosa Crespo-Rodriguez, Jan R. T. van Weering, Debby Van Dam, Peter P. De Deyn, Adrian Apetri, Liliane Schoofs, Marc H. Mercken

**Affiliations:** 1 Neuroscience department, Janssen Pharmaceutical Companies of Johnson and Johnson, 2340 Beerse, Belgium; 2 Animal Physiology and Neurobiology, KU Leuven, Naamsestraat 59, 3000 Leuven, Belgium; 3 Janssen Prevention Center, Janssen Pharmaceutical Companies of Johnson & Johnson, Archimedesweg 6, 2333 CN Leiden, The Netherlands; 4 Neurochemistry Lab, Clinical Chemistry department of the Amsterdam UMC, Amsterdam, the Netherlands; 5 Department of Clinical Genetics, Center for Neurogenomics and Cognitive Research (CNCR), Amsterdam UMC, Amsterdam, the Netherlands; 6 Laboratory of Neurochemistry and Behavior, University of Antwerp, Universiteitsplein 1, 2610 Antwerp, Belgium; 7 Department of Neurology and Alzheimer Center Groningen, University Medical Center Groningen (UMCG), Hanzeplein 1, 9713 GZ Groningen, The Netherlands; 8 Department of Neurology and Memory Clinic, Hospital Network Antwerp (ZNA) Middelheim and Hoge Beuken, Lindendreef 1, 2020 Antwerp, Belgium; 9 Biobank, Institute Born-Bunge, University of Antwerp, Universiteitsplein 1, 2610 Antwerp, Belgium

**Keywords:** Alzheimer’s disease, Tau, Tau aggregation, Aggregation, Seeding

## Abstract

**Background:**

Although several studies demonstrate prion-like properties of Tau fibrils, the effect of size in the seeding capacity of these aggregates is not fully understood. The aim of this study is to characterize Tau seeds by their size and seeding capacity.

**Methods:**

Tau aggregates were isolated from *postmortem* AD brain tissue and separated from low molecular weight species by sucrose gradient ultracentrifugation. Biochemical characterization of the different fractions was done by non-reducing Western blotting and aggregate-specific immuno-assays using in house developed anti-Tau monoclonal antibodies, including PT76 which binds to an epitope close to the microtubule-binding domain and, hence, also to K18. Seeding efficiency was then assessed in HEK293 cells expressing K18 FRET sensors.

**Results:**

We observed that upon sonication of Tau aggregates different size-distributed tau aggregates are obtained. In biochemical assays, these forms show higher signals than the non-sonicated material in some aggregation-specific Tau assays. This could be explained by an increased epitope exposure of the smaller aggregates created by the sonication. By analyzing human brain derived and recombinant (K18) Tau aggregates in a cellular FRET assay, it was observed that, in the absence of transfection reagent, sonicated aggregates showed higher aggregation induction. Preparations also showed altered profiles on native PAGE upon sonication and we could further separate different aggregate species based on their molecular weight via sucrose gradients.

**Conclusions:**

This study further elucidates the molecular properties regarding relative aggregate size and seeding efficiency of sonicated vs. non-sonicated high molecular weight Tau species. This information will provide a better knowledge on how sonication, a commonly used technique in the field of study of Tau aggregation, impacts the aggregates. In addition, the description of PT76-based aggregation specific assay is a valuable tool to quantify K18 and human AD Tau fibrils.

## Background

Despite the large heterogeneity in neurodegenerative diseases, protein misfolding and aggregation seems to be a common underlying mechanism [1]. Alzheimer’s disease (AD) is characterized by amyloidβ (Aβ) formed plaques and Tau deposits, called neurofibrillary tangles, which are the main pathological hallmarks of this disease. The presence of Tau inclusions is observed in a number of neurological diseases (Tauopathies), including AD, frontotemporal lobar degeneration with Tau inclusions, Pick’s disease, progressive supranuclear palsy, corticobasal degeneration, argyrophillic grain disease, some prion diseases, and amyotrophic lateral sclerosis/parkinsonism-dementia complex [2].

Tau-mediated toxicity is believed to be exerted by accumulation of intracellular aggregates [3] or their intermediate products [4]. As Tau pathology has been suggested to progress through a defined pattern over time [5], it is plausible that an extracellular component is contributing to the spreading of the disease [6]. Although the “Tau-spreading” hypothesis is mainly supported by indirect evidence, recent PET data have confirmed the progression through the different stages and demonstrate a significant correlation between Tau pathology and cognitive decline [7]. While the pattern of propagation and the brain regions affected by Tau pathology are identified, it is not completely understood how this mechanism of Tau spreading occurs. In addition, seeding experiments with Tau species derived from interstitial fluid and cerebrospinal fluid collected from AD individuals, suggested the presence of seeding-competent Tau species in extracellular fluids [8].

Multiple Tau seeding experiments have shown prion-like properties of aggregates isolated from Tau-containing brain. The seeding efficiency of both human AD brain and P301S mouse brain derived tau aggregates has been already demonstrated in various cellular assays [9]. Furthermore, injection models of Tau seeding have been successfully developed and include injections of P301S brain material [10], total *postmortem* Tauopathy brain homogenates [11], or high molecular weight species of Tau [12–14] in mouse brain.

There are however important differences in the structure of heparin induced recombinant Tau aggregates [15–17], which are mostly comprised of straight filaments, when comparing with AD brain-derived aggregates which have a more complex paired helical filament structure [17, 18]. There is also evidence that Tau aggregates isolated from brains of patients of different Tauopathies will lead to different strains when used in seeding experiments [19–21].

In this communication, we characterized Tau seeds from human *postmortem* AD brain by correlating an intrinsic physicochemical analysis with the assessment of functionality as competent seeds, elucidating the effects of sonication on tau aggregates. Multiple techniques were essential for this such as aggregation specific analysis using among others, an antibody specific for the MTBD region of Tau, size-based separation, and FRET-Tau analysis.

## Methods

### Animals

All in vivo experiments were conducted in strict accordance with the guidelines of the Association for Assessment and Accreditation of Laboratory Animal Care International (AAALAC), with the European Communities Council Directive of 24th November 1986 (86/609/EEC) and with protocols approved by the local Institutional Animal and Use Ethical Committee. Mice expressing the 0N4R isoform of human Tau with a P301S mutation were obtained from the MRC Laboratory of Molecular Biology, Swindon (UK) and were backcrossed on a C57bl/6 background at the JAX laboratories. Mice were single housed in an enriched environment, individually ventilated cages and under 12/12 h light/dark cycles (light at 6:00 AM). At 6 months of age, mice (*n* = 10) were sacrificed by decapitation and spinal cord was harvested and snap frozen.

### In vitro Tau aggregation

Fibrils derived from the P301L-mutated aggregation-prone repeat domain of 4R Tau (K18-P301L) [22] fibrils were prepared as previously described [23]. Briefly, myc-tagged K18-P301L Tau (Tebu Bio) (66.67 μM) was incubated in the presence of 133 μM Heparin sodium salt (MP Biomedicals) in 100 mM ammonium acetate pH 7.0 at 37 °C under gentle agitation. After 5 days, samples were centrifuged for 1 h (184,000 x g; TLA 100 rotor, Beckman) and supernatants were kept for the analysis of remaining monomeric K18. The pellet was washed twice in 1 mL PBS and finally resuspended in 400 μL PBS to obtain 333.33 μM K18 fibrils, which were directly aliquoted and frozen (− 80 °C) or frozen after a sonication step (Branson probe sonicator, amplitude 15%, total sonication time was 2 min in pulses of 2 s with 10 s interval between them). To control for temperature effects samples were on ice during sonication.

### Fibril preparation

Tau fibrils from frozen *postmortem* tissue from the frontal lobe obtained from a histologically confirmed Alzheimer patient (Braak stage VI) or from frozen spinal cord from aged P301S Tau Tg mice [24] were partially purified by a modified method of [25]. Briefly, frozen tissue was homogenized in 10 volumes of cold buffer H (10 mM Tris, 800 mM NaCl, 1 mM EGTA and 10% sucrose, pH 7.4) using a glass/Teflon Potter tissue homogenizer at 1000 rpm (IKA Works, Inc.; Staufen, Germany). The homogenate was subsequently centrifuged for 20 min at 27,000 x g. The pellet was discarded, and the supernatant was adjusted to a final concentration of 1% (w/v) N-lauroylsarcosine and incubated with rotation for 1.5 h at 37 °C. Subsequently, the extract was centrifuged at 184,000 x g for 90 min at 20 °C. The pellet was washed in PBS and resuspended in 25 times less volume of PBS, aliquoted and frozen at − 80 °C. To ensure that seeding experiments and sucrose gradient ultracentrifugations are performed with samples containing equal amounts of Tau, extracts are quantified by Western blotting to estimate the total Tau content after denaturation (Fig. S2). In addition, the PT51/PT51 aggregate-selective MSD assay was used to compare aggregated tau content [13].

Sonication was performed as described in the in vitro Tau aggregation protocol.

### Western blot

Samples (5 μL of crude paired helical filaments (PHF) fraction or 20 μL of sucrose gradient fractions) were diluted in NativePAGE™ Sample Buffer and NativePAGE™ 5% G-250 Sample Additive loaded on 3–12% native PAGE gel (Thermo Scientific) according to the manufacturer’s instructions. After the electrophoresis, the gels were incubated 20 min in Tris/Glycine/SDS transfer buffer and blotted on a polyvinylidene difluoride (PVDF) membrane. After destaining in 100% methanol to remove excess of Coomassie dye, that was present during the run, membranes were rinsed in Tris-buffered saline-Tween (TBS-T) and blocked in TBS-T containing 5% non-fat dried milk. Blots were incubated with HRPO-labelled Tau antibody (hTau10 or AT120) for 2 h at RT or with primary antibodies followed by a secondary HRPO-labelled anti-mouse antibody. Detection was done using enhanced chemiluminescence (West Dura; Thermo Scientific) using a Amersham Imager 600 RGB (GE Healthcare). Images were captured in automated exposure modus.

### CFP/YFP FRET cell analysis of aggregation

Tau fibril extracts, or their subfractions, were added to Tau FRET Biosensor cells [9] as described [13]. Samples were tested by reverse transfection (Lipofecteamine2000) or co-incubation. After 48 h or 7 days respectively, cells were harvested using trypsin. FRET analysis was performed on a BD FACSCanto II (BD Biosciences). When using reverse transfection, samples diluted in PBS are incubated in a cell culture plate with Lipofectamine 2000. Cells are then plated together with the Lipofectamine Parameters analyzed were increase in FRET signal and total number of cells.

HEK293-FRET CFP/YFP cells kindly provided by the group of Prof. Marc Diamond [9] were maintained as an adherent culture with cell culture medium Gibco™ DMEM supplemented with 10% Fetal Bovine Serum (BioWest), 1% Penicillin Streptomycin (Sigma-Aldrich), 1X GlutaMAX™ (Thermo Scientific) and sodium pyruvate (Thermo Scientific) at 37 °C, 5% CO_2_ and controlled humidity. At 80% confluency, cells were splitted twice a week by trypsinization, centrifugation at 1000 x g (Centrifuge 5810, Eppendorf), resuspension in cell culture medium and plating at a density of 1 × 10^6^ cells/flask (Fisher Scientific).

To run a functional seeding assay, HEK293-FRET CFP/YFP cells were plated into a 96 well poly-D-lysine coated (Greiner Bio-One) at a density of 2500 cells per well in a volume of 130 μL of cell culture medium and kept overnight in the incubator. On the second day, the standard curves and samples were diluted in PBS and co-incubated with the cells. Total volume of each well was adjusted to 150 μL with PBS. After 7 days of incubation, the cells were washed once with PBS and trypsinized with 50 μL for 5 min and transferred to a polypropylene 384 well-plate (Thermo Scientific) containing 30 μL of Hank’s Balanced Salt Solution (Sigma-Aldrich).

When transfection reagent was used during FRET analysis, 25 μL of Lipofectamine2000 is diluted in Opti-MEM with a total volume of 1 mL. 10 μL of the sample to be analyzed is incubated in a 96 well poly-D-lysine coated (Greiner Bio-One) plate well for 10 min. After incubation 55,000 cells diluted in 130 μL medium are added per well. After 48 h of incubation cells are collected in the same way as when not using transfection reagent. FRET analysis was performed with BD FACSCanto™ II (Becton Dickinson, New Jersey, USA using the Violet laser. The Pacific Blue channel is used to measure CFP. The AmCyan channel is used for the detection of YFP signal, but with an alternative filter set to better separate the YFP emission from the CFP emission. In this case, a 556LongPass and 585/42 BandPass filterset was used instead of the 502LP and 510/50BP filterset that is normally used in the AmCyan configuration.

Controls were done by initially gating a negative population of cells (treated with no pro-aggregating sample). Gating for positive cells was performed by treating cells with a sample known to cause aggregation a FRET signal. The same gating strategy was then always applied to each experiment and confirmed with negative and positive controls. As published in [9] Tau seeding activity is measured in homogenates from AD brain but not in the same homogenates from non-tauopathy brain. For mouse brain homogenates, it was previously shown that spinal cord homogenates from WT mice do not contain seeding activity while in the same extracts from P301S mice Tau seeding increases with age [13].

### Biochemical analysis using Meso scale discovery (MSD) ELISA

Coating antibodies PT76, PT51 [13] or AT8 [26] were diluted in PBS (1 μg/ml) and dispensed into MSD plates (30 μL per well) (L15XA, Meso Scale Discovery), which were incubated overnight at 4 °C. After washing with 5 × 200 μL of PBS/0.5%Tween-20, plates were blocked with 0.1% casein in PBS and washed again with 5 × 200 μL of PBS/0.5%Tween-20. After adding samples and standards (both diluted in 0.1% casein in PBS), plates were incubated overnight at 4 °C under gentle agitation. Subsequently, the plates were washed with 5 × 200 μL of PBS/0.5%Tween-20, and SULFO-TAG™ conjugated detection antibody (PT76) in 0.1% casein in PBS was added and incubated for 2 h at room temperature while shaking at 600 rpm. After a final wash (5 × 200 μL of PBS/0.5%Tween-20), 150 μL of 2 X buffer T (Meso Scale Discovery) was added, and plates were read with an MSD reader. Signal/background measurements were calculated by dividing the signal by a measurement where no sample was added.

### Sucrose gradient

A discontinuous sucrose gradient was prepared as following: 1 mL of 60% (w/v) sucrose in PBS was applied to the bottom of the tube, followed by 2 mL of 50, 40, 30 and 20% (w/v) sucrose, covered by 1 mL of 10% (w/v) sucrose. Samples were diluted in PBS to a total volume of 500 μL and added on top of the gradient. This was followed by centrifugation for 16 h at 200,000 g (SW 40 Ti Swinging-Bucket Rotor) at 4 °C. Fractions of approximately 500 μL were collected and aliquoted for further analysis.

### Electron microscopy

Sonicated and non-sonicated K18 samples (4 μL) were spotted on freshly glow-discharged carbon/formvar-coated EM grids in a concentration of 2 μM and left to sediment over 1 min. Excess sample was blotted off and the grid was contrasted for 1 min with 2% uranyl acetate (Polysciences Inc) in water. Grids were blotted and air-dried before imaging in a Tecnai12 Biotwin TEM (FEI/ThermoFisher) with Veleta side-mounted CCD camera (EMSIS) at 43,000 times magnification.

## Results

### Biochemical analysis of K18 (P301L) fibrils with PT76, a microtubule binding domain (MTBD)-directed Tau antibody

Synthetic Tau-fragment-derived fibrils have been shown to be very efficacious prion-like seeding agents in vitro and in vivo [9, 23, 27, 28]. In these Tau seeding models, sonication has been used to improve the seeding capacity of aggregates even further. To determine the effect of sonication on the biochemical and biophysical properties of Tau fibrils, we initiated this study by analyzing aggregates composed of recombinant myc-tagged K18 Tau fragment with a pro-aggregation P301L mutation (referred to as “K18” in this manuscript). These fibrils are exclusively composed by identical monomeric species thus any changes in functional properties caused by sonication could only be attributed to altered biophysical properties and not to differences in protein constitution. One important fact on these K18 Tau fragments is that they are only composed of 4R Tau, while Tau in human brain has multiple isoforms with 4R and 3R Tau.

**Fig. 1:**
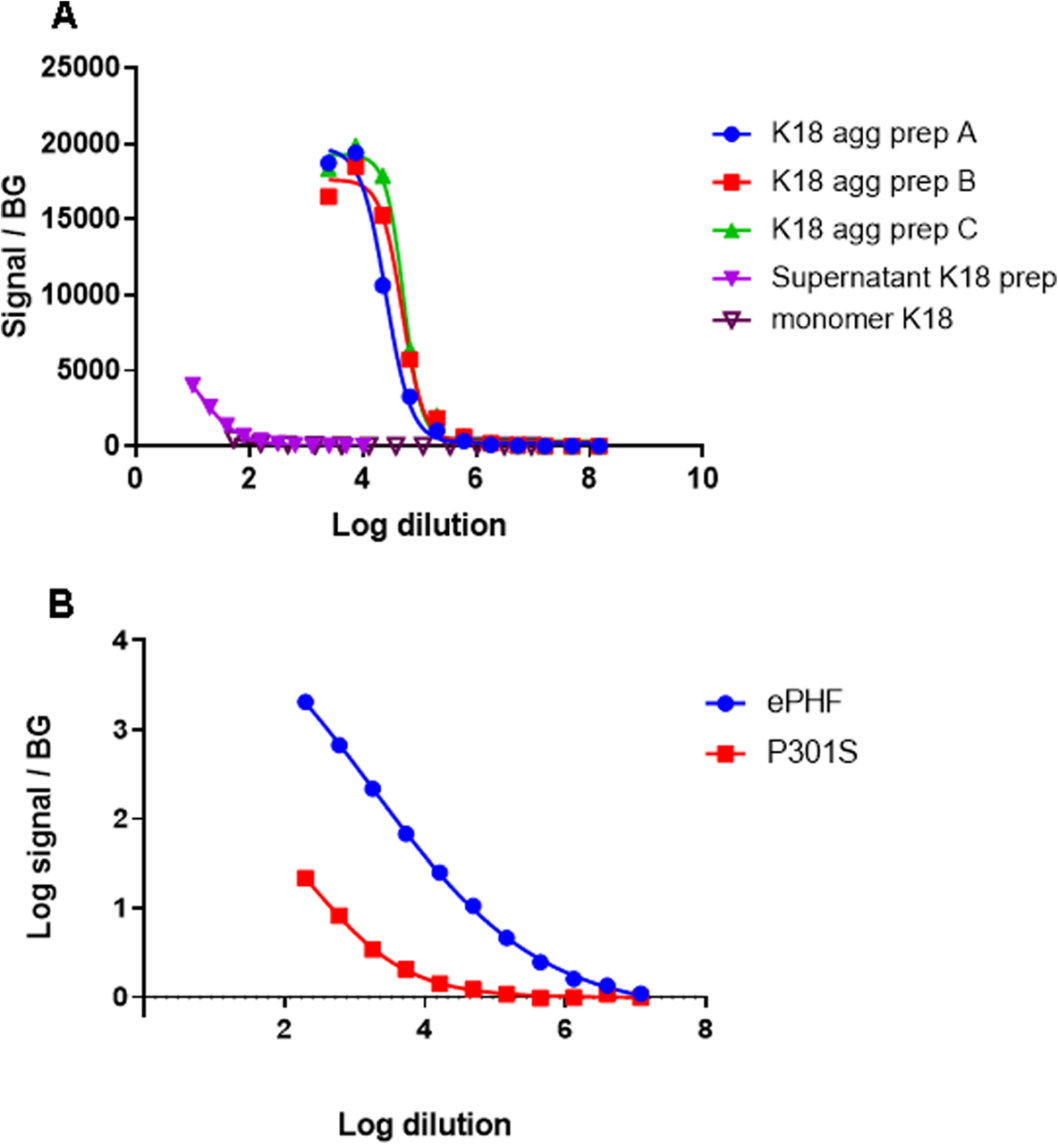
MSD analysis of recombinant and biological Tau samples. **a** Three different preparations of K18 aggregates were analyzed using a PT76/PT76 aggregation selective MSD assay and compared against a representative supernatant fraction after ultracentrifugation and against an equivalent amount of monomeric K18. **b** Comparison of PT76 aggregations specific MSD using two different biological sources, ePHF and P301S spinal cord total extracts

From our in-house antibody collection, PT76, developed by immunization with human AD brain-derived PHF as first described in Vandermeeren et al. [13] was found to bind a Tau epitope encompassing residues 249–258 which is localized N-terminal from the MTBD. Therefore, this antibody is suited to detect K18 containing samples as shown with ELISA data (Fig. S1). In addition to an MSD assay that detects linear myc-K18 proteins, an aggregation-selective self-sandwich MSD assay using PT76 as coating and as detection antibody was developed. Aggregate-selectivity of this assay was confirmed by analysis of increasing dilutions of three different K18 fibril preparations in comparison to dilutions from an equivalent amount of monomeric K18 (confirmed by native PAGE and by different dilutions of supernatant obtained after ultracentrifugation). The three independent K18 fibril preparations have consistent dilution curves (Fig. 1a). Almost no signal is observed for dilutions of the (non-aggregated) K18 preparation before fibrillization (Fig. 1a), demonstrating the aggregate-selectivity of the PT76/PT76 MSD assay. This assay did also not detect substantial signals in the supernatant after ultracentrifugation of the aggregated K18 prep which confirmed that the majority of aggregated K18 is incorporated in the pellet fraction.

Using a PT76/PT76 aggregation specific MSD on different dilutions of human AD enriched PHFs (ePHF) compared to Tau fibrils derived from P301S Tau Tg mouse brain (Fig. 1b) it was possible to observe that for preps with similar Tau content (Fig. S2), the same dilutions containing ePHF result in a much higher signal compared to P301S derived seeds. This can be caused by a lower PT76 epitope exposure in P301S seeds compared with ePHF.

### Sonication of K18- and human AD brain-derived fibrils

**Fig. 2:**
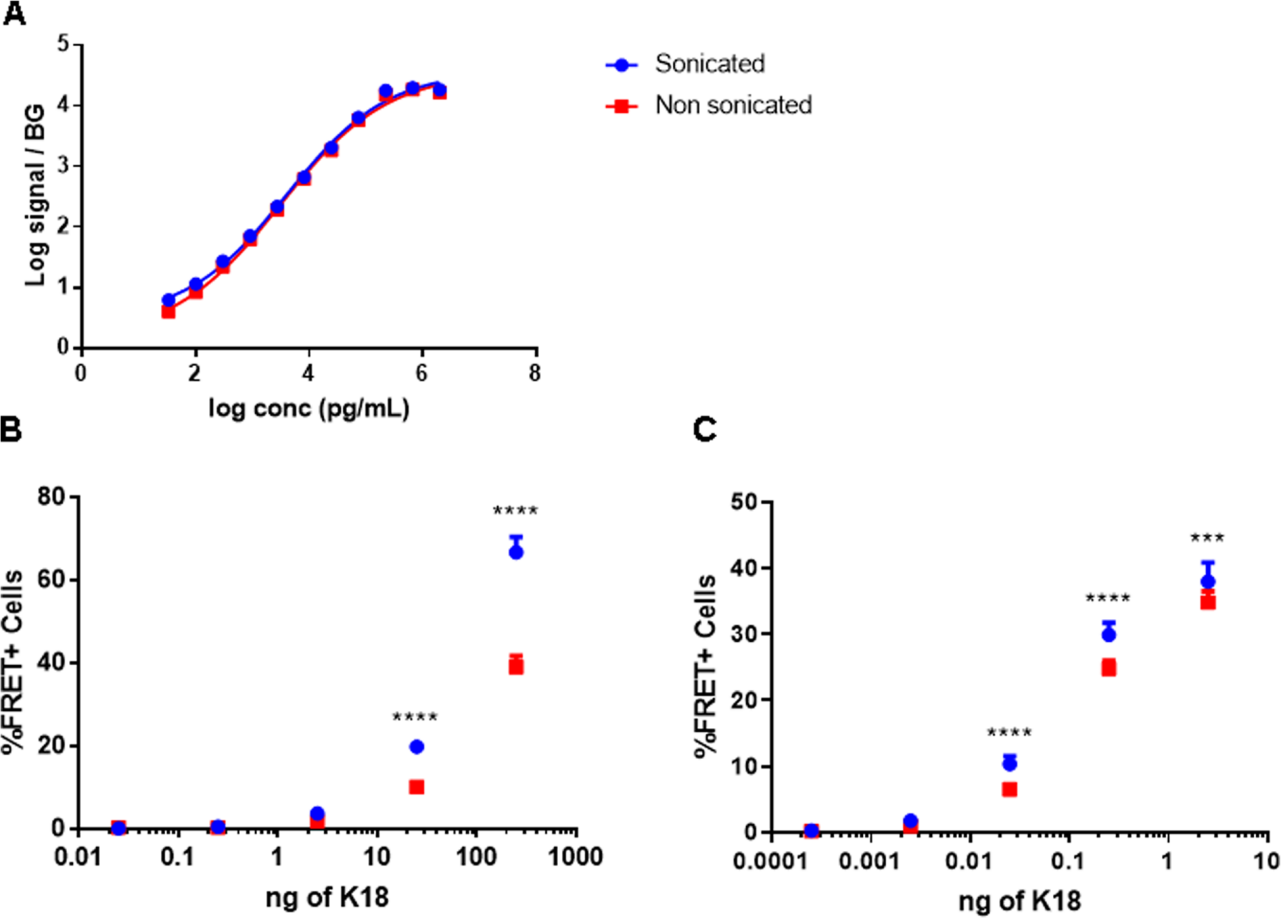
Effect of sonication on biochemical and functional analysis of K18. **a** In vitro aggregated K18 fibrils (sonicated and non-sonicated), were analyzed with a PT76/PT76 aggregation selective MSD assay. **b**, **c** Seeding capacity of K18 Tau seeds (sonicated and non-sonicated) were investigated by adding different amounts of the preparations to K18-FRET cells, in the absence (panel **b**) or presence (panel **c**) of transfection reagent. Induction of aggregation was evaluated and expressed as by percentage of FRET. (∗∗∗*p* < 0.001; ∗∗∗∗*p* < 0.0001, two-way ANOVA analysis)

Seeding experiments using Tau fibrils often involve sonication to improve efficiency [23, 29]. To better understand this phenomenon, a K18 fibril preparation was divided in two aliquots, one of which was sonicated and the other remained untreated. These samples were then analyzed with MSD and cellular assay analysis (Fig. 2). When dilution series of these samples were analyzed with a Tau aggregate-selective PT76/PT76 MSD assay, similar signals were observed in sonicated and non-sonicated samples (Fig. 2a) indicating that sonication did not alter the amount of aggregated material. Conversely, evaluation of these samples in a cellular Tau seeding assay [9] revealed a higher signal induced by sonicated K18 fibrils compared to equal amounts of non-sonicated fibrils (Fig. 2a,c). This difference was more prominent when the assay was performed in the absence of transfection reagent (Fig. 2b). Together with the PT76/PT76 MSD analysis, these data demonstrate that sonication improves the seeding potency of K18 fibrils without changing the overall exposure of PT76 epitopes. On one hand sonication of the fibrils could generate species with an altered size and improved cellular uptake. In addition, the amount of aggregates will be higher, which can lead to more events of seeding. The observed, modest, effect by sonication in % FRET positive cells in the presence of transfection reagent also points to an improved seeding capacity independent of uptake which is indeed observed with in vitro seeding experiments using recombinant fibrils (Fig. S3) and with other studies [30, 31].

**Fig. 3:**
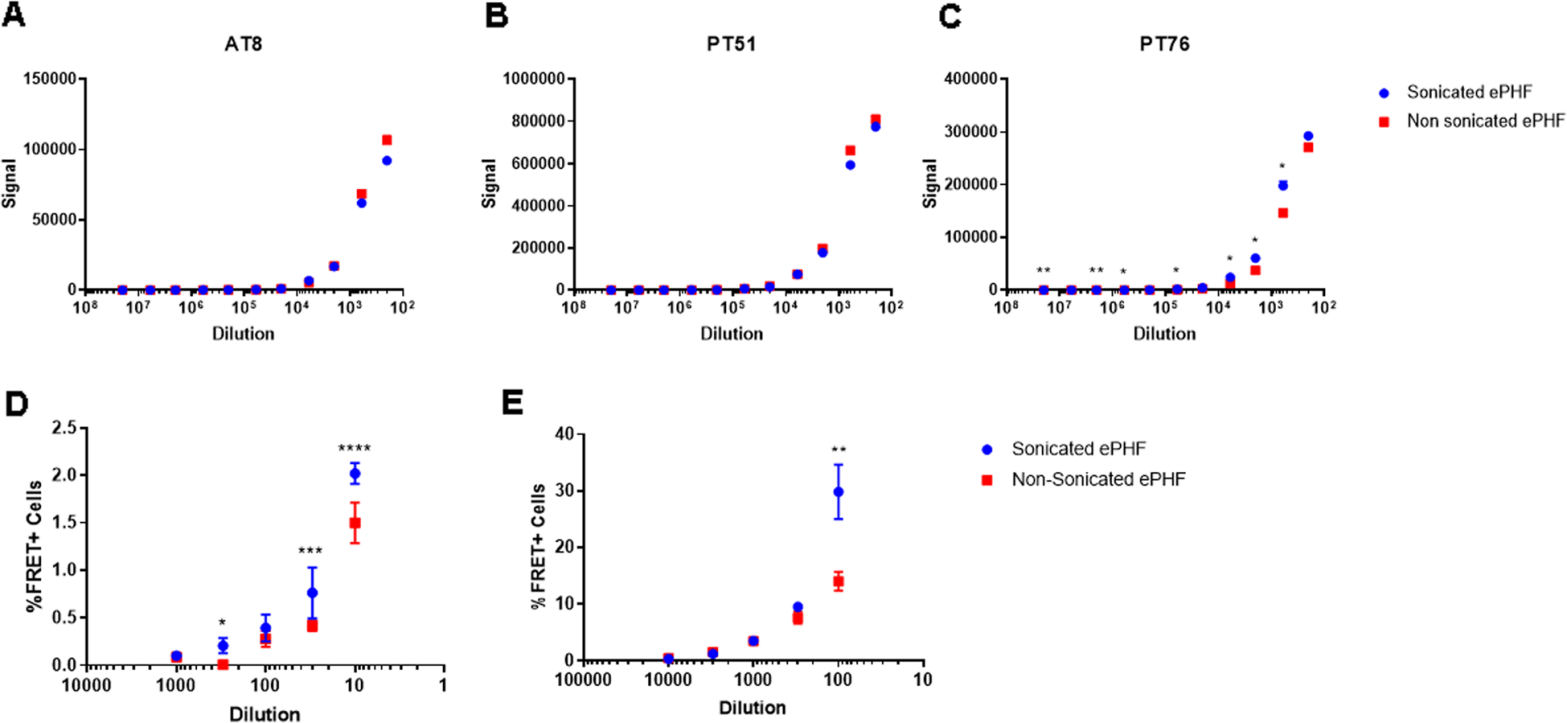
Effect of sonication on biochemical and functional analysis of human PHFs. **a**-**c** Different dilutions of human AD brain-derived PHFs (sonicated and non-sonicated), were analyzed with an AT8-AT8 (**a**), PT51-PT51 (**b**) and PT76/PT76 (**c**) Tau aggregate selective MSD assays. (D,E) Seeding capacity of PHF Tau seeds (sonicated and non-sonicated) were investigated by adding different amounts of the preparations to K18-FRET cells, in the absence (panel D) or presence (panel E) of transfection reagent. Induction of aggregation was evaluated and expressed as by percentage of FRET. (∗*p* < 0.05; ∗∗*p* < 0.01; ∗∗∗p < 0.001; ∗∗∗∗p < 0.0001, two-way ANOVA analysis)

A recent study [12] demonstrated efficient Tau seeding in WT mouse brain by human AD brain-derived PHFs prepared via a novel procedure. This procedure uses extensive sonication to resuspend the pellet after ultracentrifugation on one hand, but also additional steps to improve the purity on the other hand. To estimate the contribution of the sonication procedure to the improved seeding potency, sonicated and non-sonicated PHF preparations were analyzed both by various aggregation selective MSD assays and by cellular Tau seeding assays (Fig. 3). Self-sandwich aggregation selective assays showed similar signals for AT8 (Fig. 3a) and PT51 (Fig. 3b), and a slightly increased signal in the sonicated fraction over the non-sonicated in PT76 (Fig. 3c). Like our observed improvement in seeding potency of K18 fibrils, sonicated PHFs also appeared to be more active in the cellular seeding model. Again, this difference was more pronounced when the incubations were performed in the absence of transfection reagent (Fig. 3d, e).

### Sonication alters size distribution of K18 Tau fibrils

**Fig. 4:**
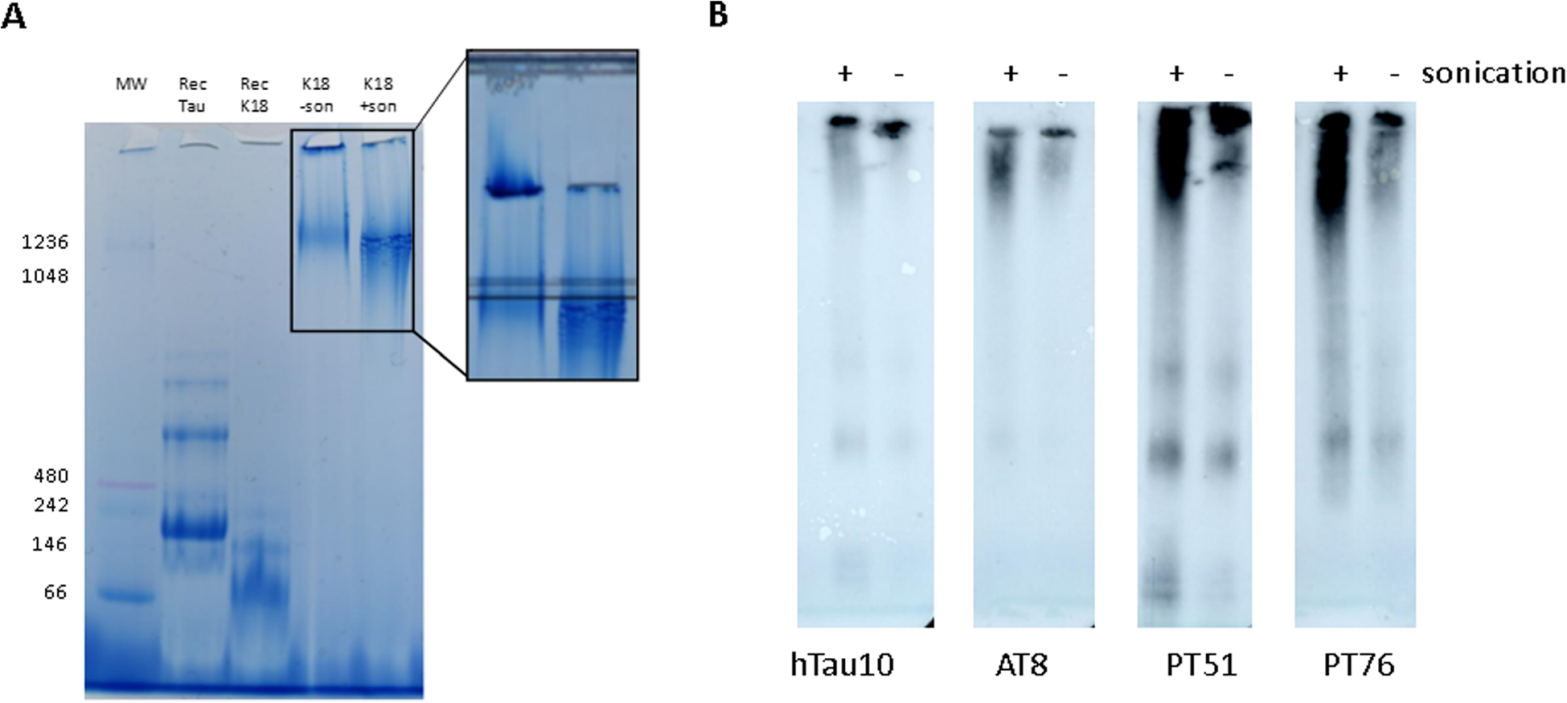
Native page analysis of K18 and PHF fibril preparations subjected to sonication. **a** In vitro aggregated K18 fibrils (sonicated and non-sonicated), non-aggregated K18 and 2N4R (WT) Tau were analyzed by native PAGE as described in materials and methods. Visualization was performed by the presence of Coomassie brilliant blue in the native PAGE sample buffer mix. The left panel shows high MW species in both aggregated K18 samples with a stronger signal for the sonicated sample. The right panel shows the same gel before cassette removal demonstrating presence of large aggregates not entering the gel. **b** Sonicated and non-sonicated PHF aggregates were analyzed by native PAGE as described in materials and methods. Detection was accomplished with hTau10, AT8, PT51 and PT76 -HRPO labelled antibodies

The data above demonstrate improved seeding activity in sonicated K18 (P301L) and human AD brain-derived Tau fibrils without changing Tau content. As this suggests involvement of alterations in biophysical properties of the fibrils, different techniques were used to demonstrate changes in size distribution induced by the sonication procedure. First, native PAGE analysis (Fig. 4) of sonicated and non-sonicated K18 fibrils revealed that most non-sonicated fibrils are too large to enter the gel, while the sonicated material migrated into the gel resulting in an increase of Coomassie signal (Fig. 4a). A similar separation of human AD brain-derived PHFs on native PAGE, detected via Western blotting using Tau antibodies binding to the N-terminus (hTau10), mid-term (PT51), phosphorylated epitope in PRD (AT8) and MTBD (PT76) [13] confirmed the reduction in size of the sonicated material (Fig. 4a). Taken together, native PAGE data provided a first indication that sonication changes the size distribution of recombinant derived fibrils and human-derived aggregates. This phenomenon was characterized in more detail using sucrose gradient ultracentrifugation to for size distribution analysis of Tau fibrils.

**Fig. 5:**
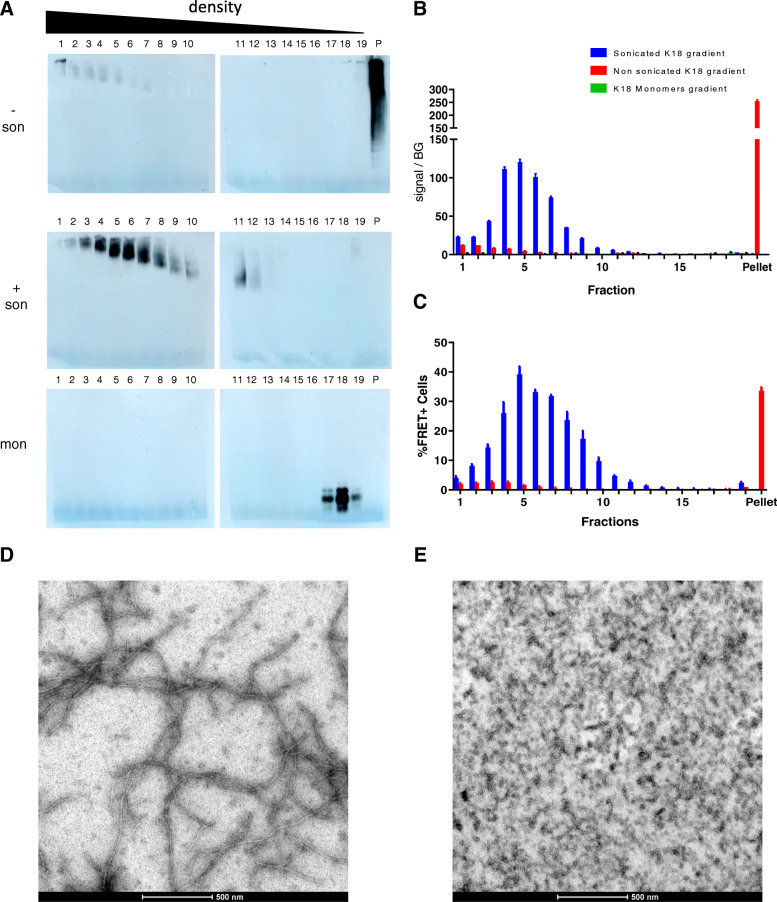
Analysis of K18 sucrose gradient ultracentrifugation fractions. Fractions obtained by sucrose gradient ultracentrifugation of aggregated, and non-aggregated K18 protein were analyzed by Western blot under native (**a**) condition. Detection performed with anti-myc primary antibody and anti-mouse HRPO-labelled secondary antibody. (Top: non-sonicated aggregated K18; middle: sonicated aggregated K18; bottom: monomeric K18). Fraction 1 has the highest density; Fraction 19 has the lowest density. Fraction P is a resuspension of the pellet in PBS. In panel (**b**), an MSD analysis of corresponding fractions using the aggregate specific PT76/PT76 assay is represented. Functional analysis of K18 sucrose gradient fractions can be observed in panel (**c**). Seeding capacity in the absence of transfection reagent was evaluated by percentage of FRET positive cells. A representative profile of at least three independent experiments is shown. K18 Tau fibrils are analyzed by electron microscopy (**d-e**). Non-sonicated (**d**) and sonicated K18 Tau (**e**) samples are used for this analysis. Images are captured at 43000x magnification

Sonicated and non-sonicated K18 fibrils and non-aggregated K18 monomers were separated by sucrose gradient ultracentrifugation and analyzed (Fig. 5). First, fractions were analyzed by native PAGE (Fig. 5a) and the blots showed that signals present in higher density fractions correspond to higher MW species. Indeed, non-aggregated K18 was only present in the least dense fractions while aggregated K18 is observed in high density fractions and in the pellet, with a density above that of 60% w/v sucrose solution. Interestingly, the signal in the pellet fraction disappeared by sonication of the fibrils. In addition, sonication altered the size distribution of K18 fibrils over the gradient with a maximum signal in the pellet and fraction 1 of the gradient of non-sonicated material to a maximal signal in fractions 4, 5 and 6 of the sonicated material.

In correspondence with these data, PT76-PT76 MSD analysis (Fig. 5b) showed that most of the aggregate signal from the non-sonicated K18 sucrose gradient migrated to the pellet, while the signal was spread out through the higher density sucrose gradient fractions of sonicated K18 fibrils, with the strongest signals in fractions 4, 5 and 6. As expected, no HMW signal was observed when analyzing sucrose gradient samples from non-aggregated K18, probing the specificity of the assay for aggregated structures. The seeding capacity of the different fractions, separated by sucrose gradients, was addressed by adding these fractions to FRET biosensor cells. In these Tau seeding experiments, it was observed that high-density fractions of the sonicated material presented at least 8-fold higher seeding capacity compared to the same fractions of the non-sonicated material (Fig. 5c). (Figure 5c) and showed a good correlation with the aggregation signal (Fig. 5b). Correlating with the aggregation MSD assay data, only the pellet fraction of the non-sonicated K18 fibrils showed efficient seeding in the biosensor cells assay. The fact that seeding was still observed in this very high-density pellet fraction might be explained by the effect of resuspension through up and down pipetting of this pellet during sample collection. This was confirmed by native Western blotting (Fig. 5a) showing that the resuspended pellet could enter the gel, while also showing lower MW fibrillary species. Nevertheless, while in the biochemical aggregation assay this pellet fraction showed the highest signal, the seeding potency of that sample remained lower in comparison to the HMW fraction 5 of the sonicated gradient. Non-aggregated K18 monomers were localized in low density sucrose gradient fractions 17, 18 and 19 and did not show seeding activity, confirming the specificity of the FRET assay.

When analyzing K18 samples by electron microscopy (Fig. 5d-e) we could observe that sonicating K18 seeds caused an alteration in the size of the fibrils from the non-sonicated samples (Fig. 5d) when compared to sonicated samples (Fig. 5e) where K18 filaments are much shorter, confirming our observations with biochemical assays and sucrose gradient experiments.

### Size separation of human AD brain- and P301S tau transgenic mouse brain-derived homogenates

**Fig. 6:**
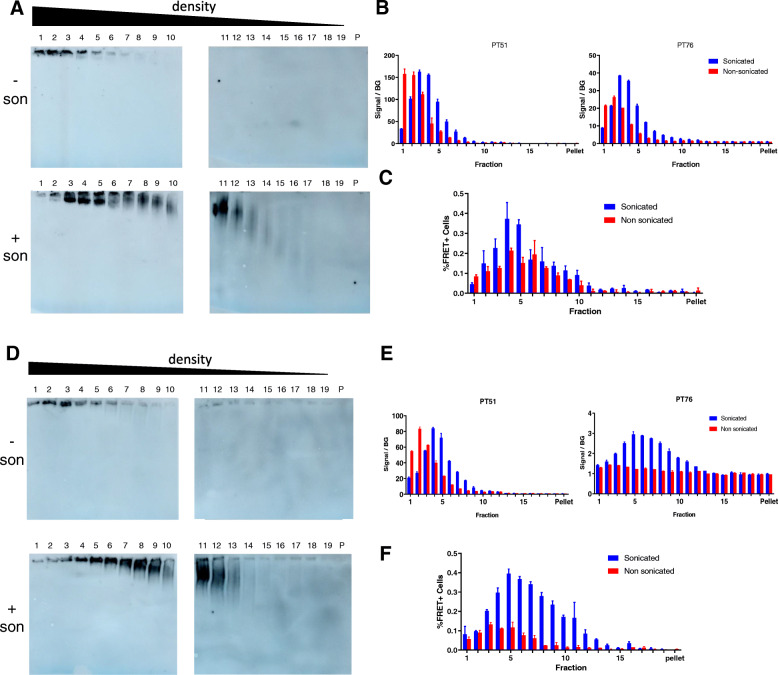
Analysis of PHF and P301S brain extract sucrose gradient fractions. Tau fibrils derived from human AD brain (**a-c**) or from P301S Tau Tg mouse spinal cord (**d**-**f**) were separated by sucrose gradient ultracentrifugation. PHF (**a**) and P301S (**d**) gradient fractions were analyzed by Western blot under native conditions. Detection performed with PT76 primary HRPO-labelled antibody. (Top: non-sonicated; bottom: sonicated). Fraction 1 has the highest density, fraction 19 has the lowest density. Fraction P is a resuspension of the pellet in PBS. An MSD analysis of PHF (**b**) and P301S fractions is performed using the indicated aggregate selective assays are represented. Functional analysis of PHF (**c**) and P301S (**f**) sucrose gradient fractions was performed by determining seeding capacity in the absence of transfection reagent (P301S) or in the presence of transfection reagent (human PHF). This was evaluated by percentage of FRET-positive cells. A representative profile of at least two independent experiments is shown

Biochemical and functional experiments demonstrated how sonication affects recombinant K18 fibrils. Next, we analyzed whether this phenomenon is also occurring in Tau seeds present in fibrils derived from *postmortem* AD brain or from P301S Tau transgenic (Tg) mouse brain [24]. In addition to this, our data already suggest structural differences between both types of fibrils apparent by a differential epitope exposure for antibodies binding close to the MTBD: i.e. PT76 and hTau21 [13]. Since the epitope exposure seems to depend on the structure and composition of the aggregate and since sonication has been shown to fragment large aggregates, it was evaluated through sucrose gradient analysis (Fig. 6) if sonication impacts size distribution and epitope exposure of Tau aggregates from human AD-brain and P301S Tg mouse aggregates.

Analysis of gradient fractions with MSD aggregation assays using antibodies PT51 and PT76 showed that non-sonicated human PHF fractions displayed high aggregate signals in the two highest-density fractions while the sonicated PHFs signals showed maximal intensity in fractions 3 and 4 (Fig. 6b). A similar shift in size distribution was observed in the sucrose gradient fractions from sonicated fibrils from P301S Tau Tg mice (Fig. 6e). This result supports the hypothesis of sonication influencing size distribution of aggregates. When functional FRET analysis of sucrose gradient fraction was performed (Fig. 6c), a modest difference between sonicated and non-sonicated Tau aggregates was observed. This can be explained by the fact that seeding of the sucrose gradient fractions was only observed in the presence of transfection reagent. In the absence of transfection reagent, fractions became too diluted to detect Tau seeding (data not shown). This indicated that postmortem human AD brain homogenates contain lower Tau seeding compared to fibrils from mutant Tau Tg mice. From data in Fig. 3d, e it was demonstrated that the effect of sonication on the seeding efficiency was less prominent when transfection reagent is used.

Interestingly, sonication of P301S brain derived aggregates increased exposure of the PT76 epitope in the sucrose gradient fractions (Fig. 6e). This is consistent with data from Vandermeeren et al., [13]. When testing the same samples on a functional analysis it was seen that sonication also increased the amount of aggregation on a shift on the fractions that are responsible for seeding for a less dense sucrose gradient fraction. Note that in this experiment the seeding could be performed in the absence of transfection reagent, a condition under which the effect of sonication is more pronounced (Fig. 3, Fig. 6f).

To understand if the observation on MSD and cellular assays are indeed due to size differences in the aggregates contained in these fractions all sucrose gradient fractions were analyzed by native PAGE and immunoblot detection with HRPO-labelled PT76. Native Page blots of sucrose gradients from sonicated extracts from human AD brain (Fig. 6a) and from P301S Tg mouse brain (Fig. 6d) displayed a shift in Tau signals when compared with the non-sonicated material, which is mostly accumulated in the first fraction. This observation comes in line with both the biochemical and functional analysis. This confirmed that sonication affects the size distribution of fibrillary Tau species in extracts from human AD brain-derived PHFs and P301S Tau Tg mice. In addition, the increased signal in some aggregation-selective assays (PT76/PT76) suggests sonication played a role in increasing epitope exposure.

## Discussion

Spreading of Tau pathology has been postulated to be a key driver in the observed spatiotemporal progression of Tauopathy-mediated neurodegeneration [5]. As this hypothesis opens the opportunity for the development of Tau immunotherapy strategies [32], the biochemical and biophysical properties of the Tau species responsible for seeding in human brain are under intensive investigation [8, 9, 12, 33, 34]. An important number of findings demonstrated the presence of Tau seeds in detergent insoluble preparations of Tau Tg mouse brain [35], but also from postmortem human Tauopathy brain [11, 12, 14]. Interestingly, the study of Guo et al. [12] describes a novel procedure to prepare Tau aggregates, which recapitulate Tau pathology upon injection in WT mice. Data from our group demonstrated Tau seeding of a sarcosyl-insoluble sample derived from *postmortem* human AD brain after injection in the hippocampus of P301L mice [13]. For both cellular and in vivo Tau seeding experiments, fibrils are sonicated to improve seeding efficiency. In this communication, we compared the size distribution and exposure of the PT76 epitope (249–258) of Tau fibrils from different sources (recombinant, Tau Tg mice brain and AD brain) and evaluated how these properties are affected by sonication.

Recently it was observed that small species of Tau have an impact on aggregation [30, 36]. Because the samples that we used to evaluate the effect of sonication or are either pre-formed aggregates or sarcosyl insoluble fractions, a limitation of this model is present. When seed-competent monomers are used the nucleation phase occurs, while after adding preformed fibrils, either recombinant or brain derived, the nucleation phase is bypassed [30, 31, 37].

After identifying and characterizing the PT76 antibody for biochemical analysis of K18 fibrils, we compared biochemical and functional properties of sonicated and non-sonicated K18-derived fibrils, prepared according to [23]. Although both samples induced seeding in the FRET biosensor cells, the sonicated K18 seeds displayed higher seeding potency and this was more pronounced in the absence of transfection reagent, thereby suggesting that sonication produces fibril fragmentation creating a higher number of available fibrils and improving seeding efficiency. The fact that high signals are observed when transfection reagent is used can be due to bigger aggregates, from which the uptake is facilitated by transfection. The fact that even under these conditions, sonication shows some improvement suggests that also the intrinsic seeding potency is increased as shown in Fig. S3 and in other studies [30, 31]. Further characterization of sonicated and non-sonicated K18 preparations by sucrose gradient ultracentrifugation experiments convincingly demonstrated that sonication induced two major alterations in the distribution of K18 aggregate species. First, the strong aggregate signal in the pellet fraction of the non-sonicated K18 fibrils was not observed in the sonicated K18 fibrils. Second, HMW species in fractions displayed higher intensities in sonicated sample when comparing with non-sonicated. Analysis of sucrose gradient with PT76/PT76 MSD confirmed the change in size distribution induced by the sonication. Also, biochemical analysis matched the observations obtained from seeding experiments with different K18 aggregate species. Surprisingly, the pellet fraction of non-sonicated K18 fibrils showed seeding potency although this was relatively low compared to its high aggregation signal in MSD. The observed seeding capacity by this pellet fraction is probably due to the resuspension of this pellet that causes limited disruption of the original large aggregate species into lower MW species. Low remaining monomer signals (< 2%) in supernatant fractions after K18 aggregation and ultracentrifugation demonstrated that the majority of K18 signal precipitated into the pellet fraction confirming that the aggregated conformation of K18 is important for the seeding potency of the aggregates. The observation that sonication will lead to a break of bigger K18 aggregates into smaller forms is further supported by the electron microscopy experiments where we could observe clear differences after sonication of K18 Tau.

To translate our findings made with K18 fibrils to in vivo-produced Tau aggregates from human AD brains, we show that sonication improves the seeding potency of these fibrils and creates smaller MW aggregates, which is indicative of a similar phenomenon as observed for K18 (P301L fibrils). Indeed, separation by sucrose gradient ultracentrifugation confirmed that sonication fragments the large aggregates in smaller fibrils with altered size distribution. Native PAGE analysis confirmed that the fibrils produced after sonication are gradually smaller, correlating with the fraction-density within the gradient. Biochemical analysis in “self-sandwich” MSD assays and cellular assays confirmed a shift in the distribution of the aggregates in the sucrose gradient fractions towards lower MW species with increased seeding potency. While PT51 detected similar signals in the sonicated and non-sonicated gradients, AT8 and PT76 showed some differences in epitope exposure after sonication, suggesting that some of these epitopes are partially shielded in the PHF structure. For PT76, previous data demonstrated that its epitope is part of the pronase-resistant core of human PHFs and even more in fibrils derived from P301S Tau Tg mice [13]. If sonication would also affect epitope exposure, the sonication-induced increased PT76 signal would be expected to be more pronounced in Tau seeds from Tg mice.

Sucrose gradient fractions from sonicated and non-sonicated fibrils from Tau Tg mice showed a shift in size distribution accompanied by an increase of seeding capacity by the sonication procedure. Although this effect was comparable to the sonication-driven changes on AD brain-derived PHFs, the increased exposure of the PT76 epitope after sonication was more drastic. Since the epitope of PT76 is proximate to the MTBD, in which the hexapeptide stretches ^275^VQIINK^280^ and ^306^VQIVYK^311^ [38] have been shown to display strong propensity for β-Sheet formation, the increased PT76 epitope exposure after sonication could increase the exposure of the above described pro-aggregation motifs, which may additionally explain the increased seeding potency of sonicated fibrils. Another possible reason for a more exposed epitope on non-recombinant seeds after sonication is a reduction of other proteins sticking to the surface of aggregates being displaced from them after sonication. This could make that both uptake by cells and binding of PT76 antibodies are increased. Since the fibril uptake in the actual experiments is not quantified, imaging studies with K18 fibrils will help to confirm the effect of sonication on the cellular uptake of Tau fibrils.

The difference in PT76 signal between P301S and human derived PHF sucrose gradient fractions also suggests that there is an intrinsic difference in these aggregates’ structure and consequent exposure to PT76. According with results from Fitzpatrick et al., [18] PT76 epitope is in a region outside the core of the aggregate. This would mean that even in high molecular weight NFT’s this epitope would be to some extent exposed. This also correlates with our previous observations using pronase [13] where PT76 epitope was more conserved in P301S aggregates after proteolysis than PHF. Finally, observations using trypsin made by Watanabe et al., [39] showed that PT76 is outside the trypsin resistant core of the aggregate, which would mean that this epitope is exposed in Tau aggregates obtained from AD patients *postmortem* material.

A study with sucrose gradient separation of P301S Tg mouse seeds [35] also demonstrated that small fibrils, but not monomeric or small oligomeric Tau species, are the most seeding-competent species in vitro and in vivo. In that study, seeding-competent species were present in the 40% sucrose gradient, while the fibrils present in the 50% sucrose fraction where no longer seeding competent. This suggests a limit to the size of the aggregates that allows cellular uptake. Also, Wu and colleagues [4] demonstrated that in cells, recombinant Tau firstly misfolds into low MW aggregates that then further assemble into fibrils. While the low MW aggregates can be taken up by neuronal cells, the long fibrils extracted from human brains were not taken up. The authors hypothesized that internalization of Tau aggregates in cultured neurons depends on the conformation and size of the aggregates, which is in accordance with the findings described here. If so, this study explains the requirement of the sonication of Tau seeds in in vitro and in vivo studies that are currently in use to investigate pre-clinical evaluation of Tau antibodies.

Small oligomeric species with potent seeding activity, as suggested by two previous studies [34, 40] were not observed, which is in accordance with previous data [13] and work from Jackson and colleagues [35] showing that short fibrils are the seeding-competent Tau species that are present in brain homogenates from P301S Tau Tg mice. Collectively, the experiments comparing sonicated and non-sonicated K18 aggregate species confirm seeding capacity of larger MW aggregates, but not of oligomers, which were not observed in the analyzed samples. Moreover, separation of human AD brain- and P301S Tau Tg mice brain-derived fibrils by means of a sucrose gradient also reveals that the major seeding competent fractions of these samples are present in high MW fractions as confirmed by native PAGE blots. Here, the sonication generates a shift in size distribution combined with an increased exposure of epitopes close to the MTBD. Previous work suggested that exposure of these epitopes can be determined by the structural alterations between recombinant fibrils and fibrils derived from tauopathy brain [17]. In addition, conformational templating assumes [21] that also between different tauopathies, fibril structure and epitope exposure can vary [18]. The knowledge of which size of aggregates is responsible for seeding and which antibodies better recognize these forms of Tau can have an impact in how antibodies are selected for therapy, not only for AD but also for other tauopathies, such as Progressive supranuclear palsy, corticobasal degeneration or frontotemporal dementia. These analyses could be applied to these different Tauopathies in order to understand in which fractions are the more toxic species of Tau and which are more recognized by certain antibodies. By understating the differences and similarities between these disorders one could find better tools to study and potentially treat them.

The results in this work also suggest that smaller species of Tau fibrils are more involved with the progression of the disease. This would mean that a potential therapy would ideally target Tau fragments in an early stage of the progression of the disease, where the big neurofibrillary tangles are not yet formed. This work provides insight in the size distribution and epitope exposure of the different Tau fibrillary species derived from different sources and how these properties correlate to their seeding potency while also giving us better tools to study Tau aggregation. In particular, the effect of sonication on recombinant and human AD brain-derived extracts, which are being used in different cellular and in vivo seeding models, is demonstrated and explained. This knowledge can provide a better understanding of the properties of the true Tau seeding components during disease progression and how to target these by immunotherapy.

## Conclusions

Taken together, this work suggest that sonication affects biochemical, biophysical and functional properties of Tau aggregates from different sources such as in vitro aggregated K18 and *postmortem* preparations from human AD brain and P301S Tau Tg mouse brain. Data provided in this manuscript supported two phenomena:

1. Sonication breaks large aggregates into smaller sized fibrils. While the effect of size distribution was very prominent for K18 fibrils, this was also observed for the aggregates derived from human and Tau Tg mouse brains.
2. Sonication increases intrinsic seeding potency in vitro which also contributes to the effect of sonication on the seeding efficiency of *postmortem* brain homogenates observed after transfection (which bypasses the limitation of cellular uptake).

## Supplementary Information

**Additional file 1 Figure S1.** Fig- ELISA analysis of a K18 P301L Tau fibril preparation supernatant fraction. **Figure S2.** Comparison of Tg P301S mouse seeds and human AD-brain seeds detection in Western blotting quantified by recombinant tau loading control (2N4R tau). **Figure S3.** Effect of sonication on seeding efficiency in vitro*.*

**Additional file 2.** Raw images.

## Data Availability

The datasets during and/or analysed during the current study available from the corresponding author on reasonable request.

## Abbreviations

AD: Alzheimer’s Disease
CFP/YFP: Cyan/yellow fluorescent protein
EGTA: Ethylene glycol-bis(β-aminoethyl ether)-N,N,N′,N′-tetraacetic acid
FRET: Fluorescence resonance energy transfer
HRPO: Horseradish peroxidase
MSD: Meso Scale discovery
MTBD: Microtubule-binding domain
MW: Molecular weight
PAGE: Polyacrylamide gel electrophoresis
PBS: Phosphate buffered saline
PET: Positron emission tomography
PHF: Paired-helical filament
PVDF: Polyvinylidene fluoride
RT: Room temperature
Tg: Transgenic
WT: Wildtype
