# Supplementary material for: Comparison of size distribution and (Pro249-Ser258) epitope exposure in in vitro and in vivo derived Tau fibrils
Source: BMC Mol Cell Biol. 2020 Nov 12;21:81. doi: 10.1186/s12860-020-00320-y (PMC7661158; doi:10.1186/s12860-020-00320-y)

**Comparison of size distribution and (****Pro249-Ser258) epitope exposure in *in vitro* and *in vivo* derived Tau fibrils**

**André Marreiro^1,2^, Kristof Van Kolen^1,*^, Cristiano Sousa^1^, Liesbet Temmerman^2^, Bruno Vasconcelos^1^, Rosa Crespo Rodriguez^3, 4^, Jan R.T. van Weering^5^, Debby Van Dam^6^, Peter P De Deyn^6-9^, Adrian Apetri^3^, Liliane Schoofs^2^, Marc Mercken^1^**

^1^ Neuroscience department, Janssen Pharmaceutical Companies of Johnson and Johnson, 2340 Beerse, Belgium

^2^ Animal Physiology and Neurobiology, KULeuven. Naamsestraat 59, 3000 Leuven, Belgium.

^3^ Janssen Prevention Center, Janssen Pharmaceutical Companies of Johnson & Johnson, Archimedesweg 6, 2333 CN, Leiden, The Netherlands.

^4^ Neurochemistry Lab, Clinical Chemistry department of the Amsterdam UMC

^5^ Dept. of Clinical Genetics, Center for Neurogenomics and Cognitive Research (CNCR), Amsterdam UMC

^6^ Laboratory of Neurochemistry and Behavior, University of Antwerp, Universiteitsplein 1, 2610 Antwerp, Belgium

^7^Department of Neurology and Alzheimer Center Groningen, University Medical Center Groningen (UMCG), Hanzeplein 1, 9713 GZ Groningen, The Netherlands

^8^ Department of Neurology and Memory Clinic, Hospital Network Antwerp (ZNA) Middelheim and Hoge Beuken, Lindendreef 1, 2020 Antwerp, Belgium

^9^ Biobank, Institute Born-Bunge, University of Antwerp, Universiteitsplein 1, 2610 Antwerp, Belgium

*** Corresponding author**

**Figure S1 Fig- ELISA analysis of a K18 P301L Tau fibril preparation supernatant fraction.**

K18 (P301L) tau (1 mg/mL) is fibrillized and after the ultracentrifugation, the supernatant fraction was kept for analysis of remaining K18 monomers. This was measured by a MYC/PT76-HRPO sandwich ELISA on different dilutions of supernatant (SN, blue curve) compared to different amounts of monomeric K18 (green curve) as calibrant. Plates were coated with mouse anti-MYC (produced in house). After washing and blocking using 0.1 % casein, wells were incubated with indicated dilutions of SN or monomeric K18 (a). Panel (b) shows the conversion into concentration of monomeric K18. Immunocaptured K18 was detected using HRP-labelled PT76 and a TMB peroxidase EIA substrate kit (BioRad) according to the manufacturers’ instructions. Data are expressed as signal – background (n=2) from a representative experiment. The background value is determined by the signal obtained in the absence of K18. From graphs in panel (a) it can be observed that signals in SN were only measured in low-diluted samples. Calculation using the calibration curve in panel (b) revealed that from the 1mg/mL K18 applied in the reaction, only 20.9 (± 7.68) µg/mL (about 2%) remained in the supernatant.


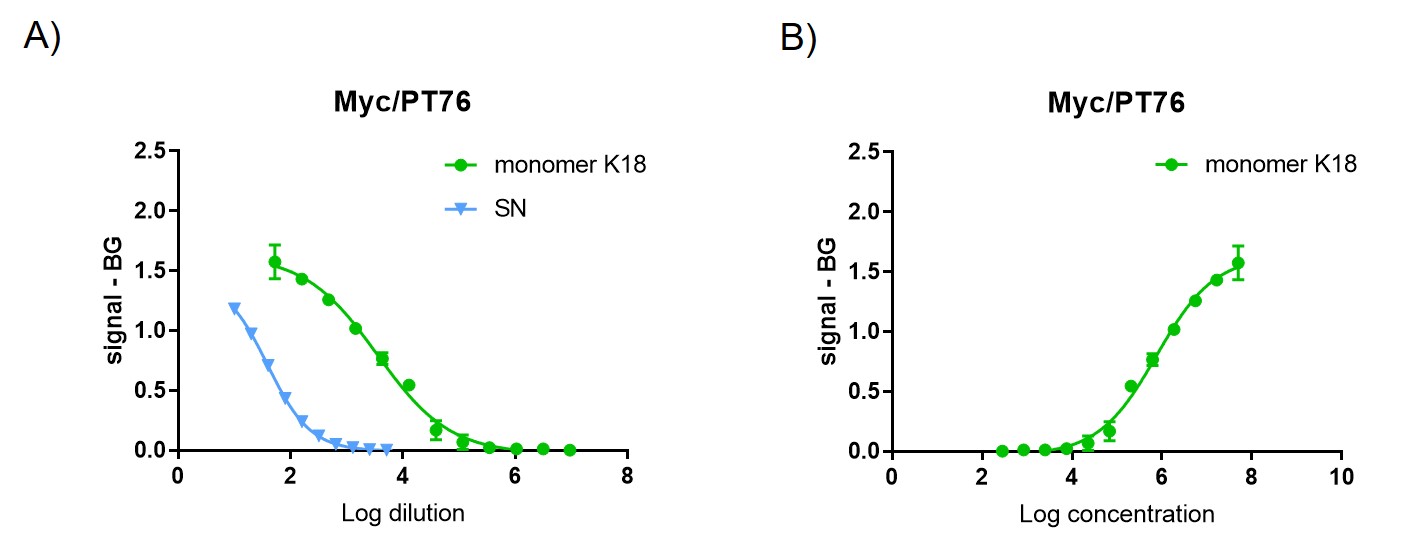


**Figure S2 – Comparison of Tg P301S mouse seeds and human AD-brain seeds detection in Western blotting quantified by recombinant tau loading control (2N4R tau).**

(A) Fluorescent hTau10 - Alexa 555 (represented with green fluorescence) Western Blot total tau quantification of tau seeds derived from P301S tau Tg mouse brainstem (3.83 µL (1) or 0.96 µL (2)) and spinal cord (3.93 µL (3, 5) or 0.96 µL (4)) or from human AD-brain (3.83 µL (6) or 0.96 µL (7)). Indicated amounts of 2N4R tau are used as calibration curve. Extracts 3/4 and 5 are from different pools of spinal cords. Bands indicated with a, b and c,d,e are indicative for an equal amount of tau present in the different aggregate preps. (B) This is confirmed by analysing different dilutions of P301S Tau and AD brain-derived PHFs with the aggregation selective PT51/PT51 MSD assay.

**
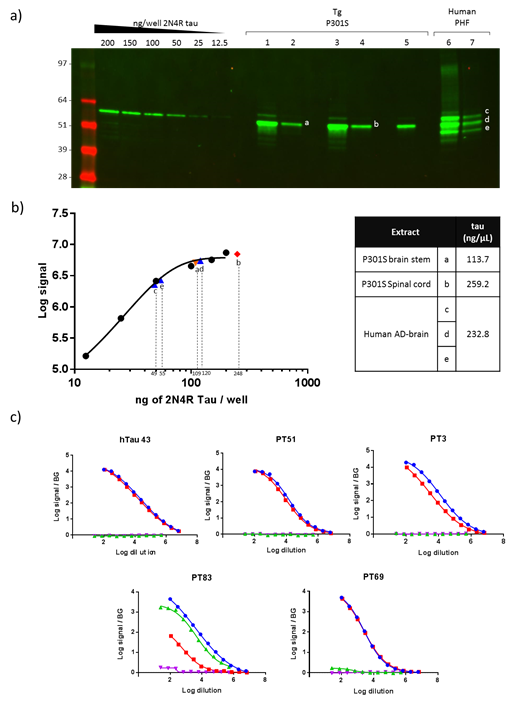
**A)

B)

**Fig S3 Effect of sonication on seeding efficiency *in vitro***

Analysis of in vitro aggregation of recombinant full length tau in the absence (black curve) or presence of pre-formed tau fibrils (different colours indicate the percentage) was performed as described in <https://www.jove.com/t/58570/in-vitro-assay-for-studying-aggregation-tau-protein-drug>. Graphs A and B show the real-time measurements in without or with sonication respectively. From these curves, it can be observed that in the presence of 2.5 % of tau seeds (dark blue curve, panel A) fluorescence intensity reached a maximum after 5h while after sonication this can be reached in the same timeframe in the presence of 0.16% or tau seeds.

1. B)


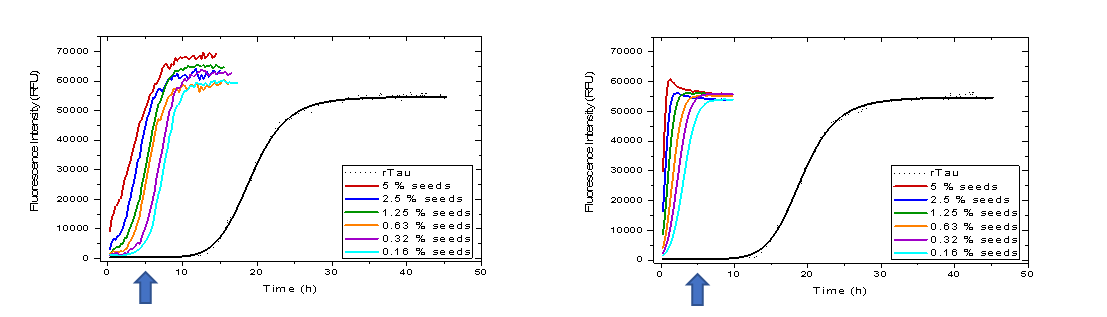

Supplement: Supplementary file 1 — Additional file 1 Figure S1. Fig- ELISA analysis of a K18 P301L Tau fibril preparation supernatant fraction. Figure S2. Comparison of Tg P301S mouse seeds and human AD-brain seeds detection in Western blotting quantified by recombinant tau loading control (2N4R tau). Figure S3. Effect of sonication on seeding efficiency in vitro. [file 12860_2020_320_MOESM1_ESM.docx]
