# Supplementary material for: Comparison of size distribution and (Pro249-Ser258) epitope exposure in in vitro and in vivo derived Tau fibrils
Source: BMC Mol Cell Biol. 2020 Nov 12;21:81. doi: 10.1186/s12860-020-00320-y (PMC7661158; doi:10.1186/s12860-020-00320-y)
Supplement: Supplementary file 2 — Additional file 2. Raw images. [file 12860_2020_320_MOESM2_ESM.docx]

**Raw images**

**Figure 4A. Native page analysis of K18 preparations subjected to sonication.** (A) In vitro aggregated K18 fibrils (sonicated and non-sonicated), non-aggregated K18 and 2N4R (WT) Tau were analyzed by native PAGE as described in materials and methods. Visualization was performed by the presence of Coomassie brilliant blue in the native PAGE sample buffer mix. The left panel shows high MW species in both aggregated K18 samples with a stronger signal for the sonicated sample. The right panel shows the same gel before cassette removal demonstrating presence of large aggregates not entering the gel. Lane 1: Native PAGE marker; lane 2: recombinant 2N4R tau; lane 3: monomeric recombinant K18 (P301L); lanes 4 and 6: aggregated non-sonicated K18 (P301L); lanes 5 and 7: (sonicated K18 P301L aggregates).


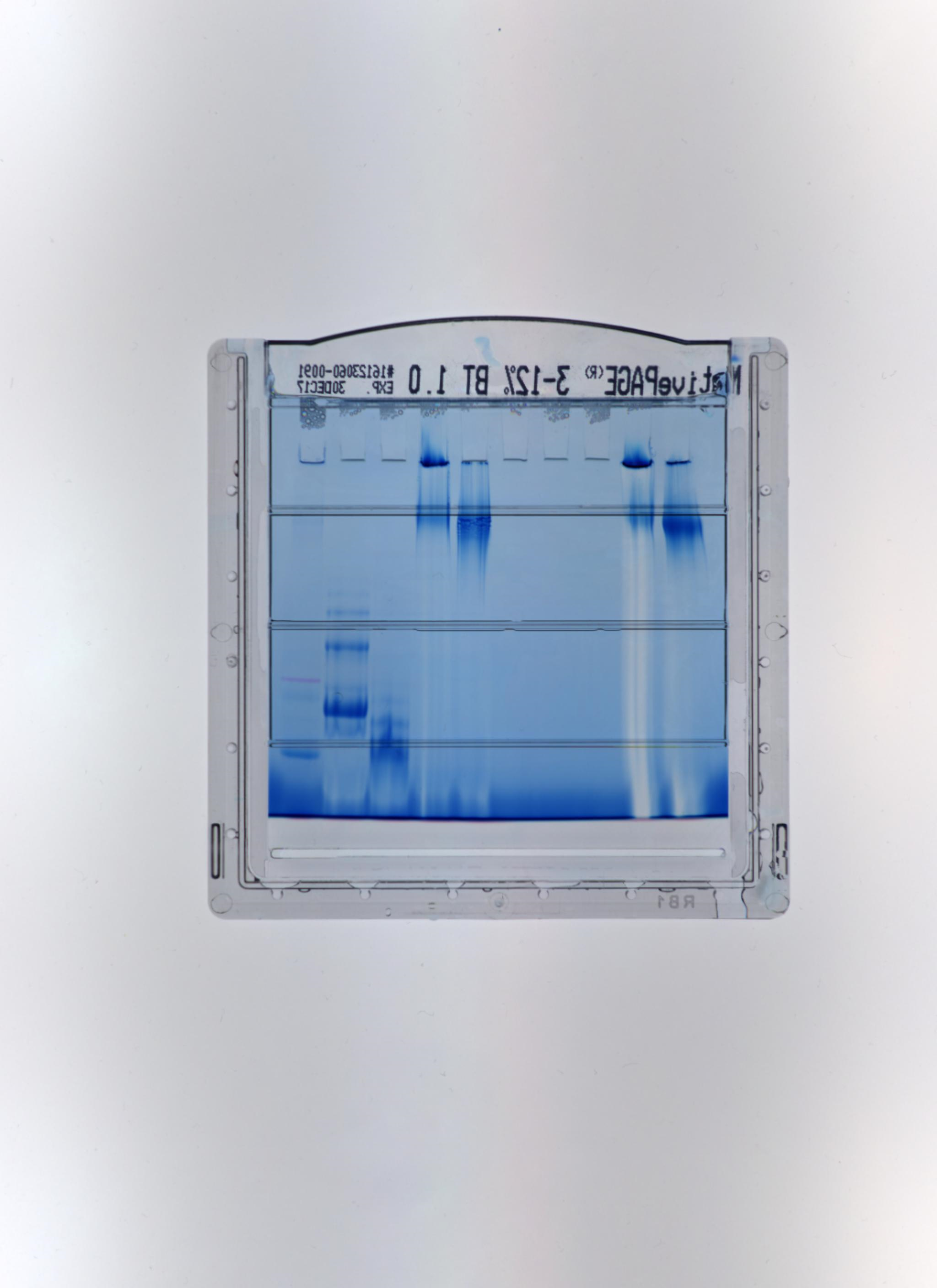


**1 2 3 4 5 6 7**

**1 2 3 4 5 6 7**


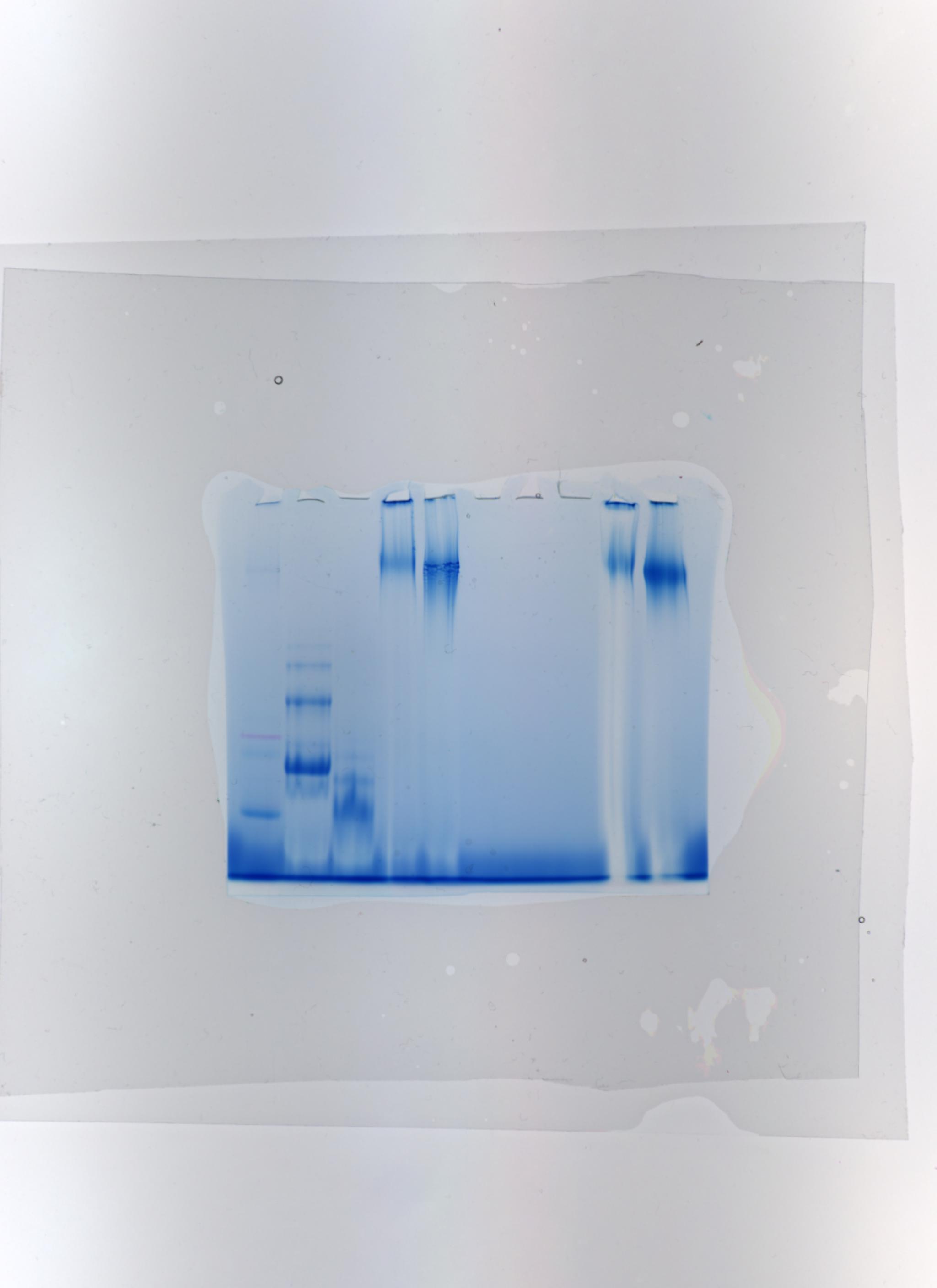


**Fig 4B Native page analysis of PHF preparations subjected to sonication** (B) Sonicated (+) and non-sonicated (-) PHF aggregates were analyzed by native PAGE as described in materials and methods. Detection was performed with hTau10, AT8, PT51 and PT76 -HRPO labelled antibodies as indicated below. The PT51 blot has been developed separately.


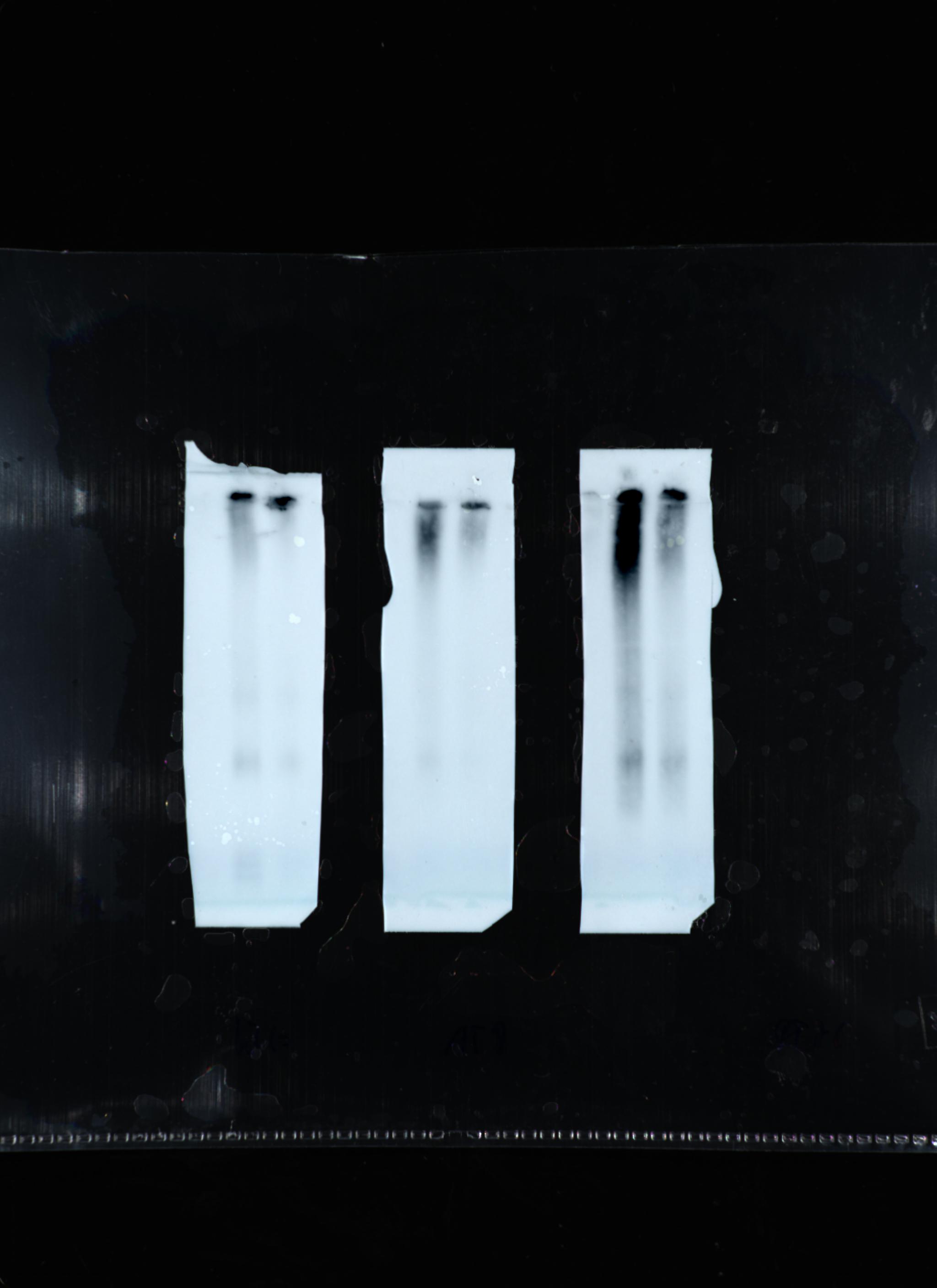

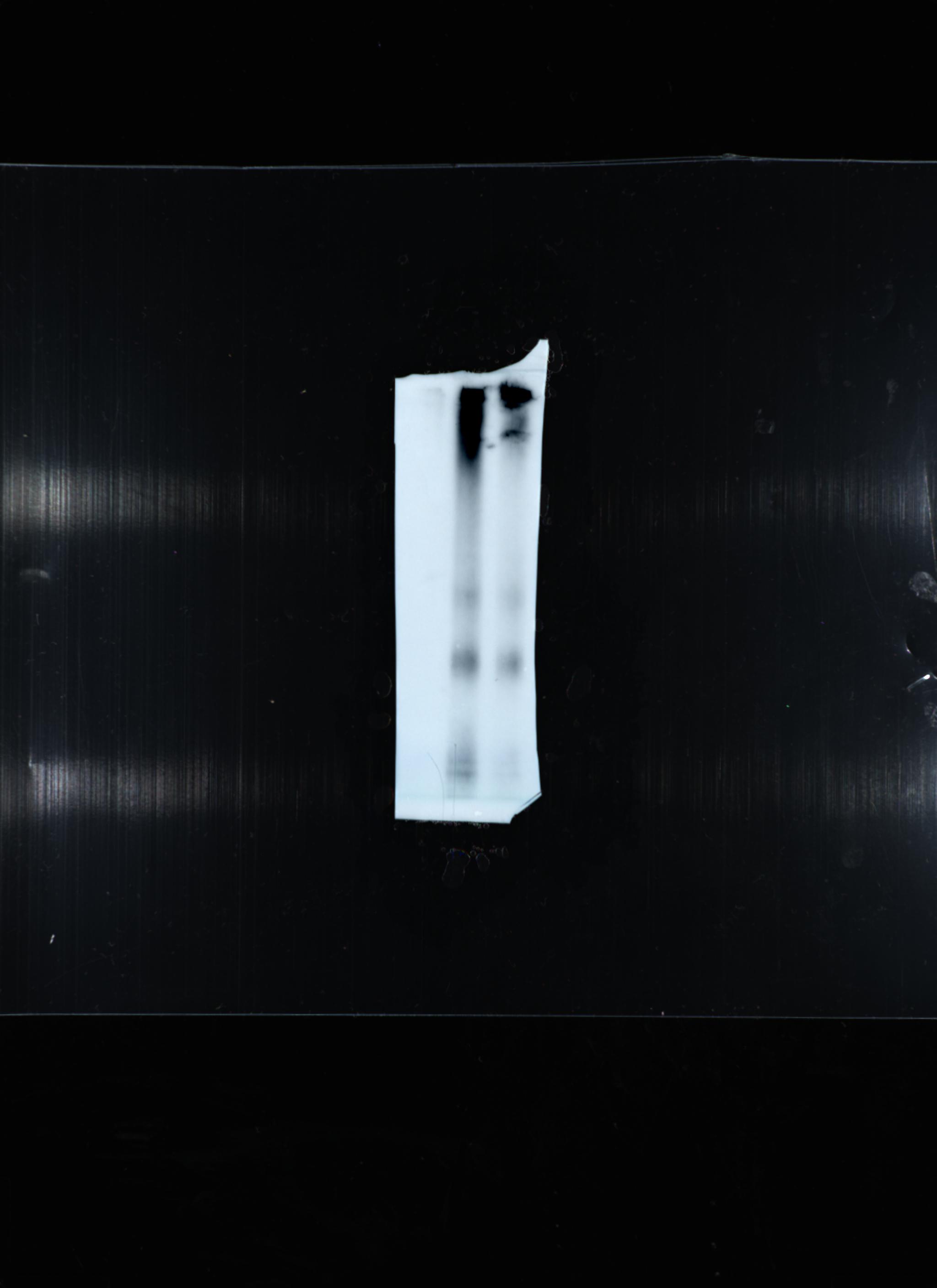


hTau10 AT8 PT76 PT51

+ - + - + - + - sonication

**Fig 5 A.** Native Western blot analysis of sucrose gradient ultracentrifugation fractions of aggregated (non-sonicated, sonicated) and non-aggregated K18 protein. Detection performed with anti-myc primary antibody and anti-mouse HRPO-labelled secondary antibody. Fraction 1 has the highest density; Fraction 19 has the lowest density. Fraction P is a resuspension of the pellet in PBS.

*Picture 5A. 1* contains simultaneous development of 2 blots with fractions 1-10 of the sonicated (top) and non-sonicated (bottom) K18 fibrils.

**1 2 3 4 5 6 7 8 9 10**


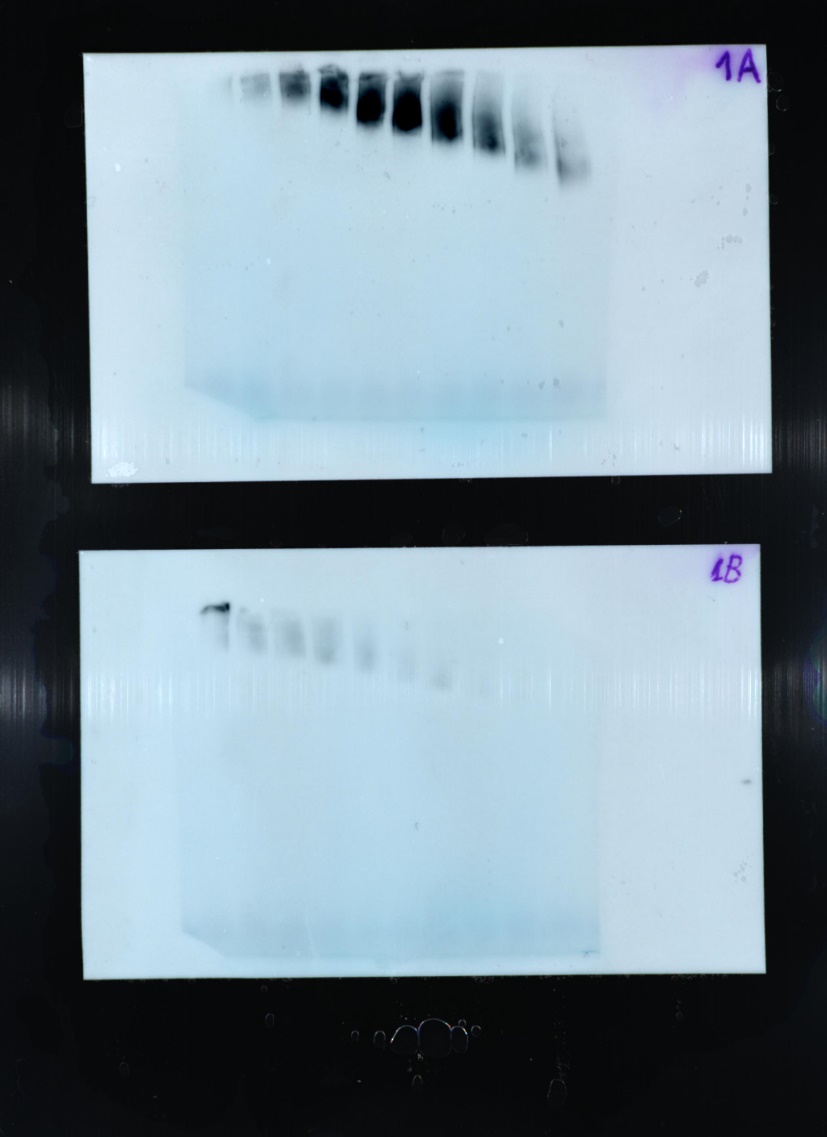


**1 2 3 4 5 6 7 8 9 10**

*Picture 5A. 2* contains simultaneous development of 2 blots with fractions 11-19 and pellet (P) of the sonicated (top) and non-sonicated (bottom) K18 fibrils.


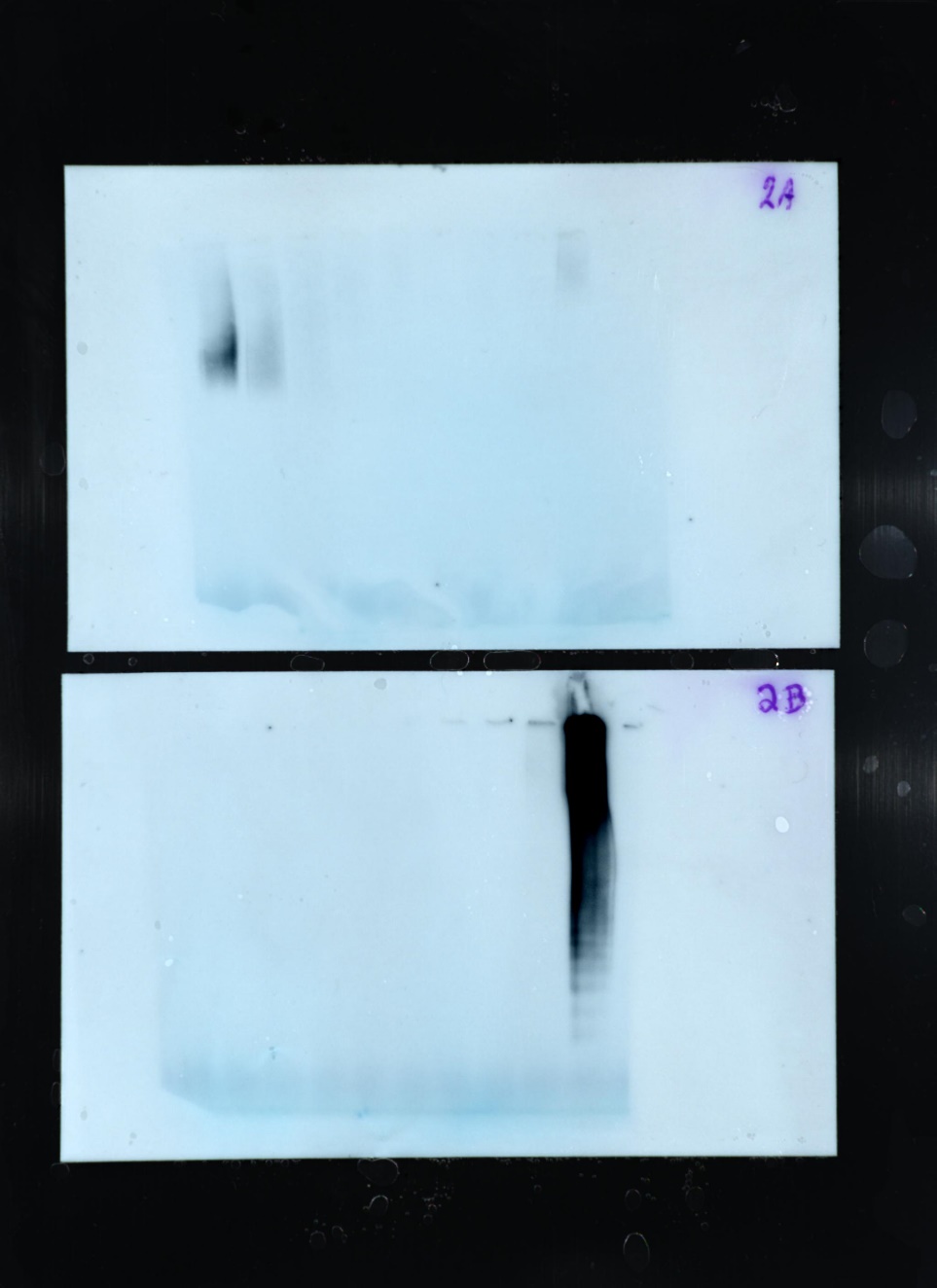


**11 12 13 14 15 16 17 18 19 P**

**11 12 13 14 15 16 17 18 19 P**

*Picture 5A. 3* contains simultaneous development of 2 blots with fractions 1-10 and 11-19 and pellet (P) of monomeric K18.


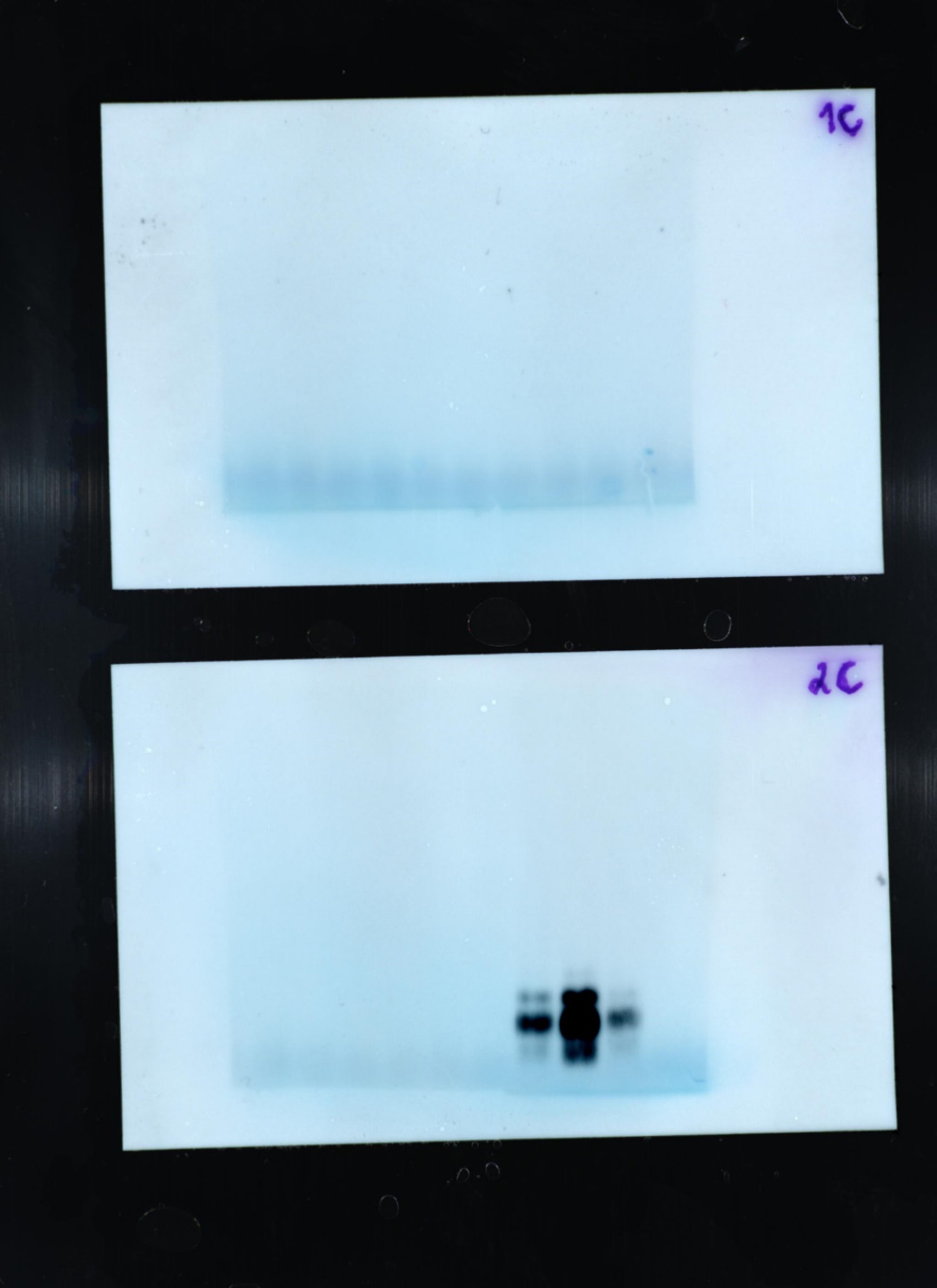


**11 12 13 14 15 16 17 18 19 P**

**1 2 3 4 5 6 7 8 9 10**

**Figure 6 A. Analysis of PHF extract sucrose gradient fractions.** Tau fibrils derived from human AD brain were separated by sucrose gradient ultracentrifugation. Gradient fractions were analyzed by Western blot under native conditions. Detection performed with PT76 primary HRPO-labelled antibody. (Top: sonicated; bottom: non-sonicated). Fraction 1 has the highest density, fraction 19 has the lowest density. Fraction P is a resuspension of the pellet in PBS.

*Picture 6A. 1* contains simultaneous development of 2 blots with fractions 1-10 of the sonicated (top) and non-sonicated (bottom) human AD PHF preps.

**1 2 3 4 5 6 7 8 9 10**


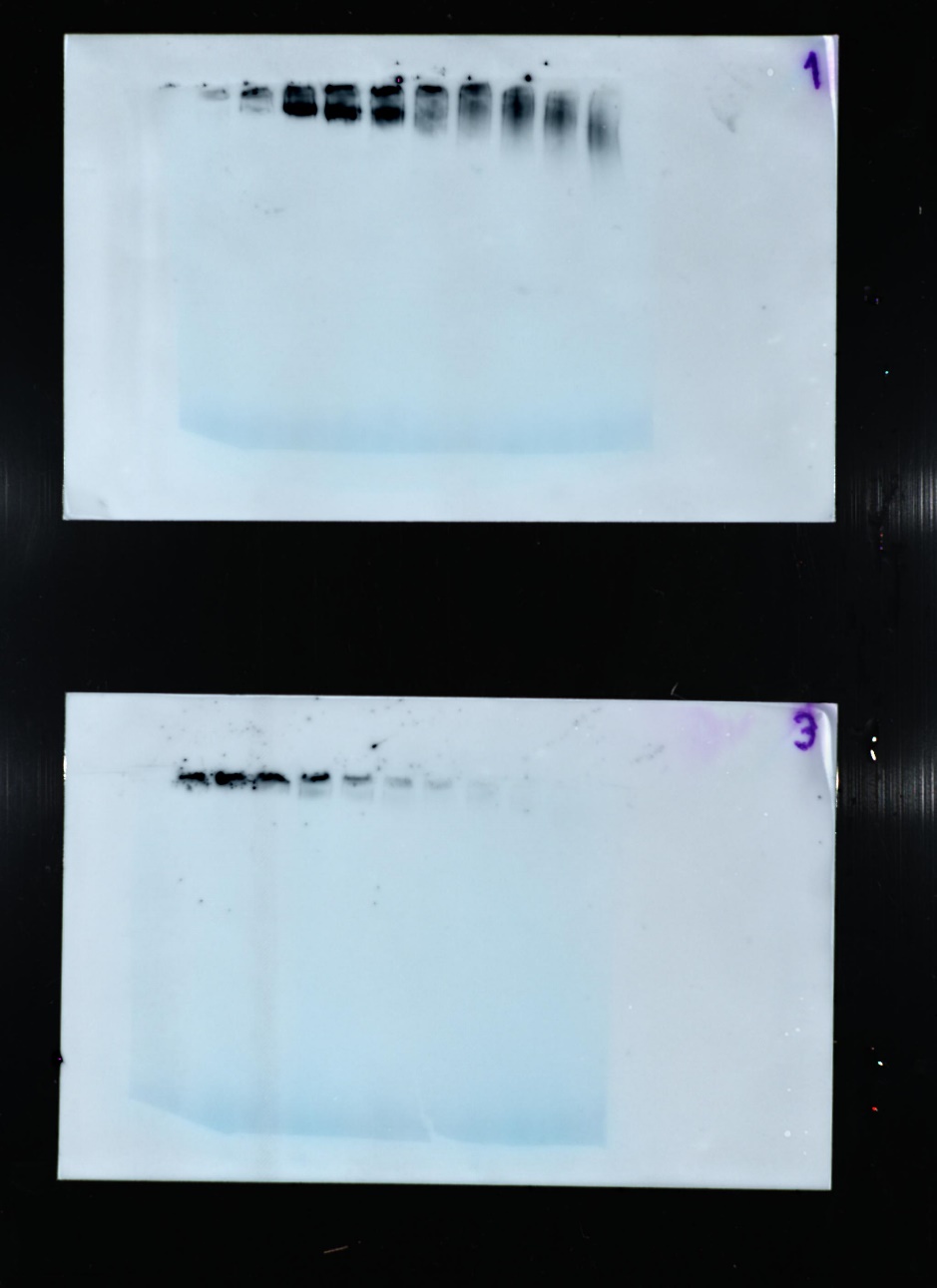


**1 2 3 4 5 6 7 8 9 10**

*Picture 6A. 2* contains simultaneous development of 2 blots with fractions 11-19 and pellet of the sonicated (top) and non-sonicated (bottom) human AD PHF preps.


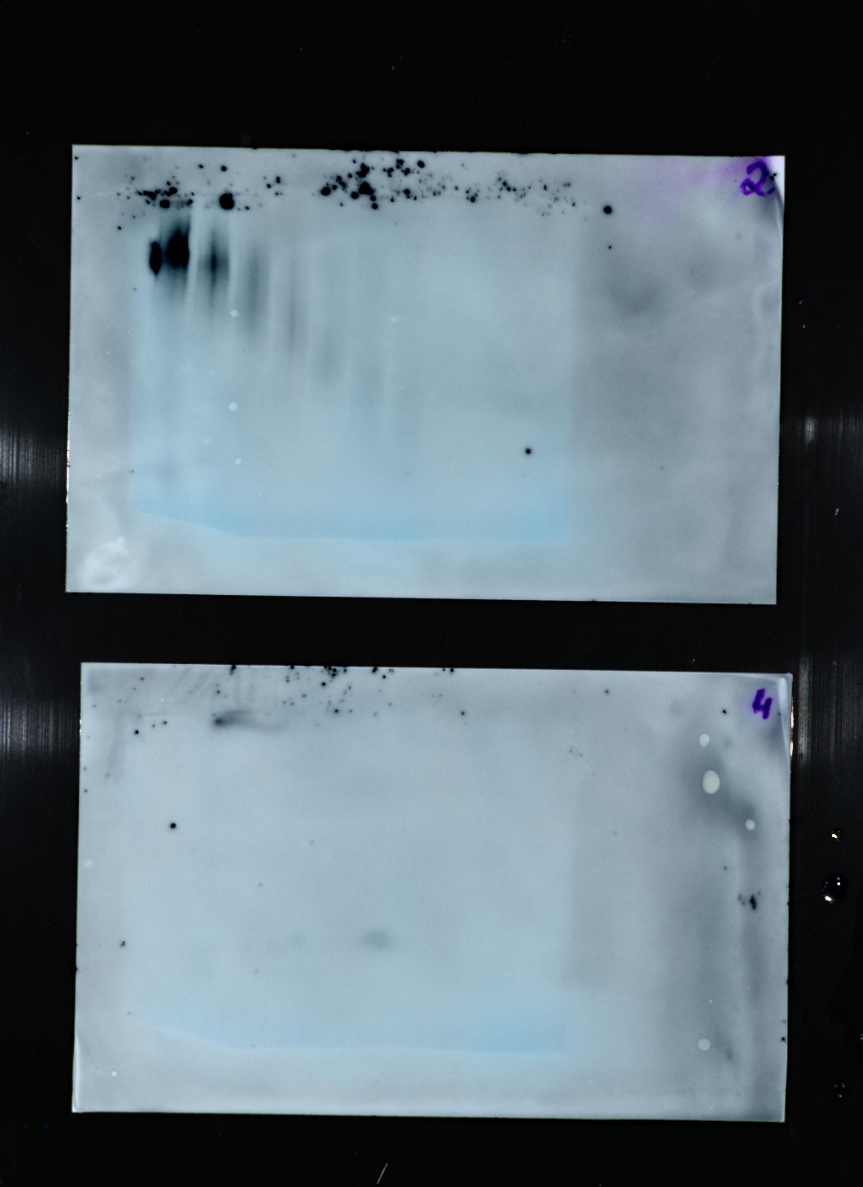


**11 12 13 14 15 16 17 18 19 P**

**11 12 13 14 15 16 17 18 19 P**

**Figure 6 D. Analysis of Tau Tg mouse spinal cord extract sucrose gradient fractions.** Tau fibrils derived from P301S Tau Tg mouse spinal cord were separated by sucrose gradient ultracentrifugation. Gradient fractions were analyzed by Western blot under native conditions. Detection performed with PT76 primary HRPO-labelled antibody. (Top: sonicated; bottom: non-sonicated). Fraction 1 has the highest density, fraction 19 has the lowest density. Fraction P is a resuspension of the pellet in PBS.

*Picture 6D. 1* contains simultaneous development of 2 blots with fractions 1-10 of the sonicated (top) and non-sonicated (bottom) P301S Tau fibrils.


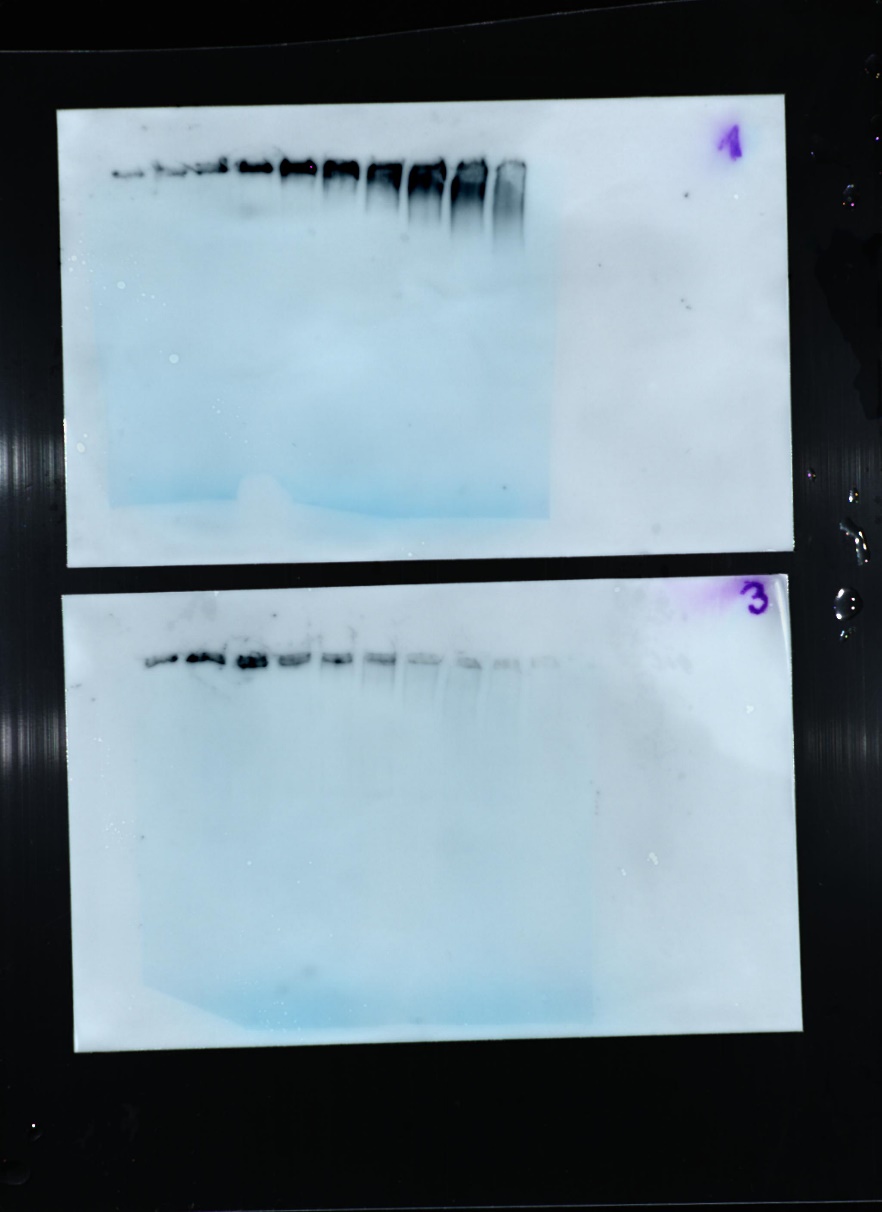


**1 2 3 4 5 6 7 8 9 10**

**1 2 3 4 5 6 7 8 9 10**

*Picture 6A. 2* contains simultaneous development of 2 blots with fractions 11-19 and pellet of the sonicated (top) and non-sonicated (bottom) P301S Tau fibrils.


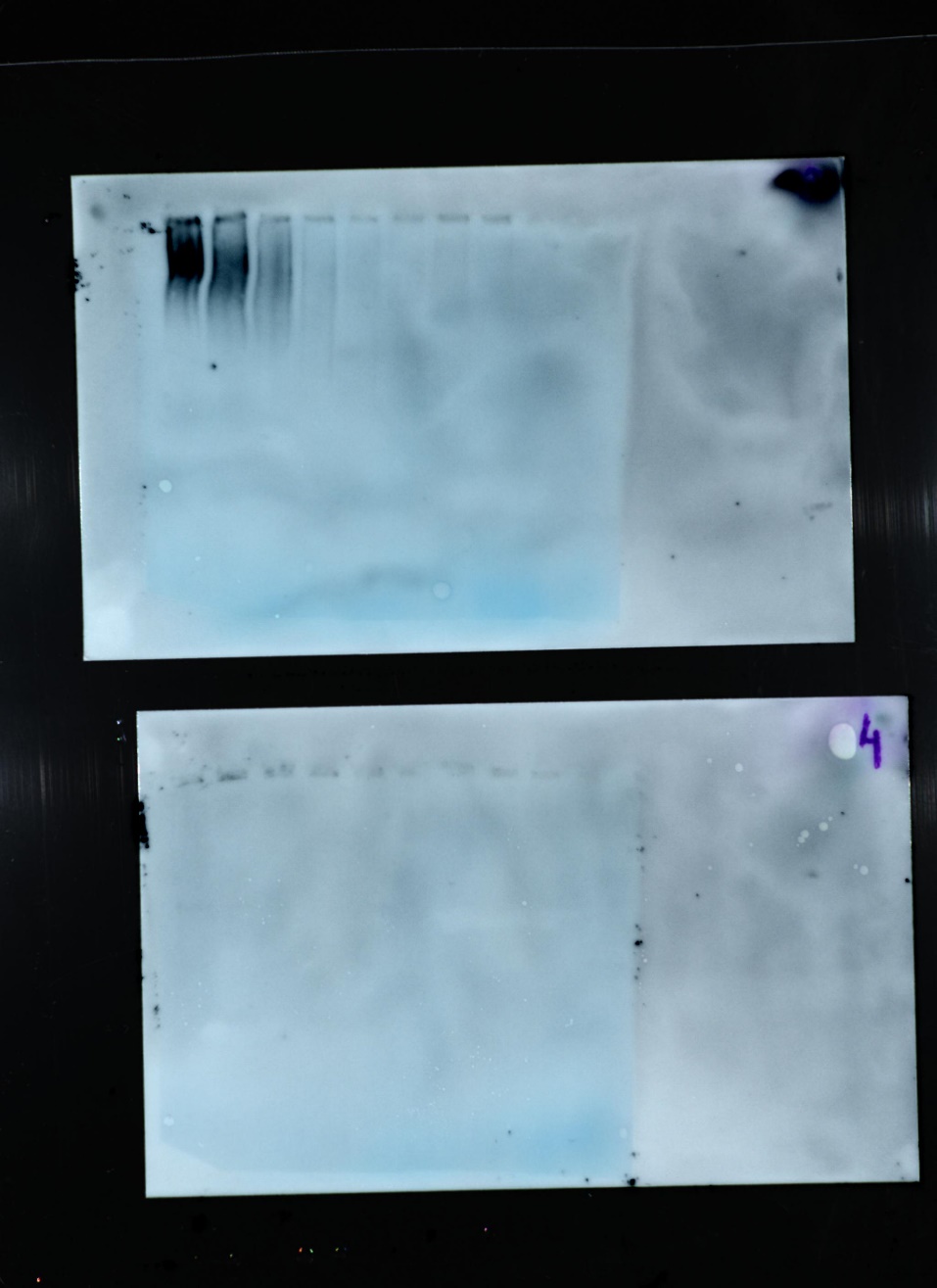


**11 12 13 14 15 16 17 18 19 P**

**11 12 13 14 15 16 17 18 19 P**
